# Specific functions for Mediator complex subunits from different modules in the transcriptional response of *Arabidopsis thaliana* to abiotic stress

**DOI:** 10.1038/s41598-020-61758-w

**Published:** 2020-03-19

**Authors:** Tim Crawford, Fazeelat Karamat, Nóra Lehotai, Matilda Rentoft, Jeanette Blomberg, Åsa Strand, Stefan Björklund

**Affiliations:** 1 Umeå Plant Science Centre, Department of Plant Physiology, Umeå University, Umeå, 901 87 Sweden; 2 Department of Medical Biochemistry and Biophysics, Umeå University, Umeå, 901 87 Sweden; 3 Present Address: Institute for Biochemistry and Biology, University of Potsdam, Potsdam, Germany

**Keywords:** Plant stress responses, Gene expression, Gene regulation, RNA sequencing, Genetics

## Abstract

Adverse environmental conditions are detrimental to plant growth and development. Acclimation to abiotic stress conditions involves activation of signaling pathways which often results in changes in gene expression via networks of transcription factors (TFs). Mediator is a highly conserved co-regulator complex and an essential component of the transcriptional machinery in eukaryotes. Some Mediator subunits have been implicated in stress-responsive signaling pathways; however, much remains unknown regarding the role of plant Mediator in abiotic stress responses. Here, we use RNA-seq to analyze the transcriptional response of *Arabidopsis thaliana* to heat, cold and salt stress conditions. We identify a set of common abiotic stress regulons and describe the sequential and combinatorial nature of TFs involved in their transcriptional regulation. Furthermore, we identify stress-specific roles for the Mediator subunits MED9, MED16, MED18 and CDK8, and putative TFs connecting them to different stress signaling pathways. Our data also indicate different modes of action for subunits or modules of Mediator at the same gene loci, including a co-repressor function for MED16 prior to stress. These results illuminate a poorly understood but important player in the transcriptional response of plants to abiotic stress and identify target genes and mechanisms as a prelude to further biochemical characterization.

## Introduction

Heat, cold, salinity or drought, constitute abiotic stress conditions that are sub-optimal for plant growth^1^. Plants have evolved complex signaling transduction pathways to perceive and respond to environmental changes. These are initiated from multiple sites within the cell and terminate in the nucleus, influencing gene expression via networks of transcription factors (TFs), allowing plants to regulate their energy expenditure and growth as they mount an adaptive response to the stress^2,3^. While many components of these pathways have been elucidated, much remains unclear about the underlying mechanism of transcriptional regulation. Phytohormones, including jasmonic acid (JA), ethylene (ET), salicylic acid (SA) and abscisic acid (ABA), play key roles in the regulation of stress responses^4,5^. Recent evidence indicates extensive crosstalk between these pathways and those of growth-regulating hormones auxin, brassinosteroid (BR), cytokinins and gibberellic acid (GA). Signals mediated by reactive oxygen species (ROS), Ca^2+^ and metabolites also play critical roles in abiotic stress responses, relaying the status of the chloroplast and mitochondria through retrograde signaling to the nucleus to influence gene expression^6,7^.

Transcriptional control of abiotic stress responses is orchestrated through a network of more than 1,500 TFs in *Arabidopsis*^8,9^. Transcriptional regulation in eukaryotic cells requires interplay of various factors; including RNA polymerase II (pol II), general transcription factors (GTFs), transcriptional activators/repressors, and co-regulators, such as Mediator^10^. Mediator is a large multi-subunit complex that interacts with promoter-bound TFs and pol II and functions as a regulatory hub to integrate inputs from different signaling pathways^11,12^. Mediator was first described in yeast^13,14^ and later found to be essential for pol II-dependent transcriptional regulation in all type of eukaryotes, including plants, based on biochemical purification and comparative genomics^15–17^.

Plant and mammalian Mediators are composed of more subunits (25–35) than yeast (21). Plant genomes contain paralogous genes for Mediator subunits; however, the secondary structures of most subunits are highly conserved^18–20^. The Mediator subunits are organized into a core, including the head, middle and tail modules, plus a dissociable cyclin kinase module (CKM)^21^. There are also four plant-specific subunits (MED34-MED37) which have so far not been assigned to any module, although MED36 was recently confirmed as a middle module subunit^22^.

Genetic analyses revealed that Mediator subunits are involved in different stress-response pathways. In particular, MED16 and MED25 are involved in multiple abiotic stress responses^23^. The *med16/sfr6* mutant displayed decreased freezing tolerance and impaired cold-induced expression of C-REPEAT/DRE BINDING FACTOR 1 (CBF1) target genes, identifying MED16 as an essential co-activator for this TF^24–26^. Two other Mediator subunits, MED14 and MED2, also regulate cold stress responses^27^. MED25 has been identified as a key component of stress-response signaling pathways in plants and interacts with multiple TFs^28,29^. MED25 links the JA receptor COI1 with chromatin and pol II via the MYC2 in response to JA signaling, and *med25* was described as sensitive to salt stress but resistant to drought^30–32^. Mediator was further implicated in plant abiotic stress responses by direct physical interaction between MED18 and NUCLEOPORIN85 (NUP85)^33^. Both *nup85* and *med18* displayed hypersensitivity to ABA and salt stress as well as overlapping defects in expression of specific stress target genes. Indeed, MED18 is intimately connected with ABA signaling, and interacts with ABI4 and YY1 to regulate expression of key abiotic stress response genes^34,35^. However, while prior research has implicated Mediator subunits as components of myriad signaling pathways, few studies have analyzed the functional role of Mediator in abiotic stress responses in detail.

Here, we use RNA sequencing (RNA-seq) to identify common target genes for short- and long-term responses of *Arabidopsis* to three types of abiotic stress – heat, cold and high salt concentrations – as well as the regulatory *cis-*elements corresponding to TF-families required for these responses. We reveal how expression of these key target genes in early stress response is affected in *med9*, *med16*, *med18*, and *cdk8*, representing subunits from each of the middle, tail, head and kinase modules, respectively. We identify possible interactions between each subunit and TFs in promoters of abiotic stress-response genes. These findings suggest key roles for specific subunits of Mediator in integration of signaling pathways during plant abiotic stress responses. In particular, we observe dysregulated transcription of key stress-response genes in the mutants during cold stress, which appear to show distinct mechanisms of activation or repression. These data provide the first systems-level evaluation of regulation of abiotic stress-responsive transcription by the plant Mediator.

## Results

### Mediator mutant lines, abiotic stress experiments and RNA sequencing

**Figure 1:**
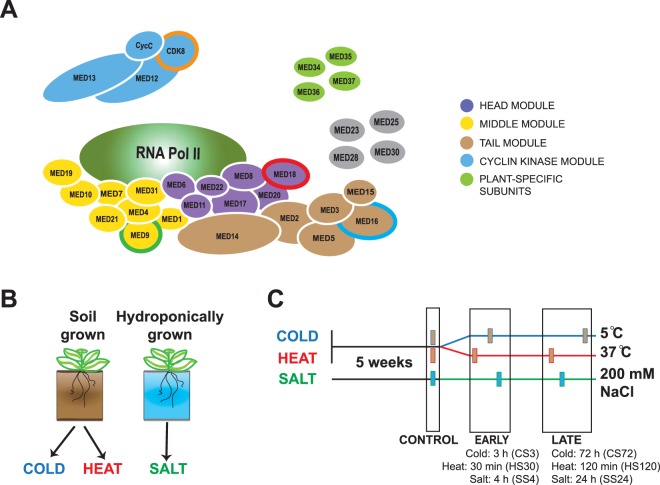
Mediator mutants and abiotic stress conditions investigated in this work. (**A**) Model of the plant Mediator complex, based on the cryo-EM and crystal structures of yeast and human Mediator and known subunit composition of the purified *Arabidopsis* complex^17,109^. Protein subunits within each of the four structural modules are coloured as follows: Head: purple; Middle: yellow; Tail: brown; Cyclin kinase: blue. T-DNA knockouts of the circled subunits (MED9, MED16, MED18 and CDK8) were selected for use in this work. Note that the localisation of the plant-specific subunits (MED34-37) within the complex is unknown, as are the positions of MED23, MED25, MED28 and MED30. In addition, the presence of the MED1 subunit has not been confirmed in the plant Mediator complex. (**B**) Setup and sampling regime for the abiotic stress experiments. Two separate populations of plants were grown for these experiments, one in soil and one in a hydroponic system as described^96^. (**C**) These plants were grown to 5 weeks old in short-day greenhouse conditions, and control samples harvested from each genotype in each population. Plants were then shifted into abiotic stress conditions: either heat (37 °C) or cold stress (5 °C) for the soil-grown plants and salt stress (fresh media supplemented with 200 mM NaCl) for the hydroponic-grown plants. Samples were harvested at an early (30 min heat (HS30), 3 h cold (CS3) or 4 h salt stress (SS4)) or a late time-point of stress exposure (120 min heat (HS120), 72 h cold (CS72) or 24 h salt stress (SS24)). Four independent biological replicates were taken for each sample, where one biological replicate consisted of one rosette leaf each from six individual plants.

To investigate Mediator function in stress responses, we selected one *Arabidopsis* mutant to represent each of the four Mediator modules (Fig. 1A). Therefore, we selected mutants of the MED9, MED16, MED18 and CDK8 subunits to represent the middle, tail, head and cyclin kinase modules, respectively. As additional criteria, we selected subunits for which mutants were available as T-DNA lines, that were likely not essential for growth (based on experiments in *Arabidopsis* and other organisms), and which did not have expressed paralogues. (Fig. 1A). We noticed that the *Arabidopsis* genome contains a potential MED9 paralogue (MED9b; AT1G29580); however, this gene encodes a truncated protein which lacks the N-terminal half and exon 3 of MED9. In addition, it is not expressed in leaves at any developmental stage according to TAIR^36^ and we could not detect any MED9b transcripts in any of our RNA-seq experiments. Homozygosity and reduced gene expression in the *med9*, *med16*, *med18* and *cdk8* mutants were confirmed using PCR and RT-qPCR, respectively (Supplemental Fig. S1A–C). Previous reports indicated flowering-time phenotypes in *med16*, *med18* and *cdk8*^37–39^, so we grew our plants in soil or a hydroponic system to mature rosette stage under non-inductive short-day conditions in order to avoid effects caused by differences in flowering time between different lines. We observed no major differences in development, although the mutants generally appeared smaller: *cdk8* and especially *med18* displayed reduced rosette diameter and biomass, while *med9, med18* and especially *med16* accumulated less total chlorophyll than Col-0 (Supplemental Fig. S2A–D).

For stress experiments, plants were sampled in control conditions (CON or CON_SS) before stress exposure (see Methods and Fig. 1B). For each stress, we sampled rosette leaves at the indicated time points (Fig. 1C) and verified induction of appropriate stress-response marker genes using RT-qPCR (Supplemental Fig. S3A–C). We confirmed that no stress-induced phenotypes were observed even at the late time-points (see Supplemental Fig. S3D for salt stress). Similar results were observed for the heat and cold stress experiments. Total RNA was isolated and sent for RNA-seq, generating an initial population of between 13–32 million reads per sample). Sequencing reads were mapped to the Araport11^40^ reference genome. We detected a background of high-confidence transcripts (with at least 2 read counts in 2 samples) for 25,914 genes in our dataset.

### Global transcriptome analysis reveals large-scale transcriptional reprogramming in response to abiotic stress and stress-specific dysregulation in mediator mutants

**Figure 2:**
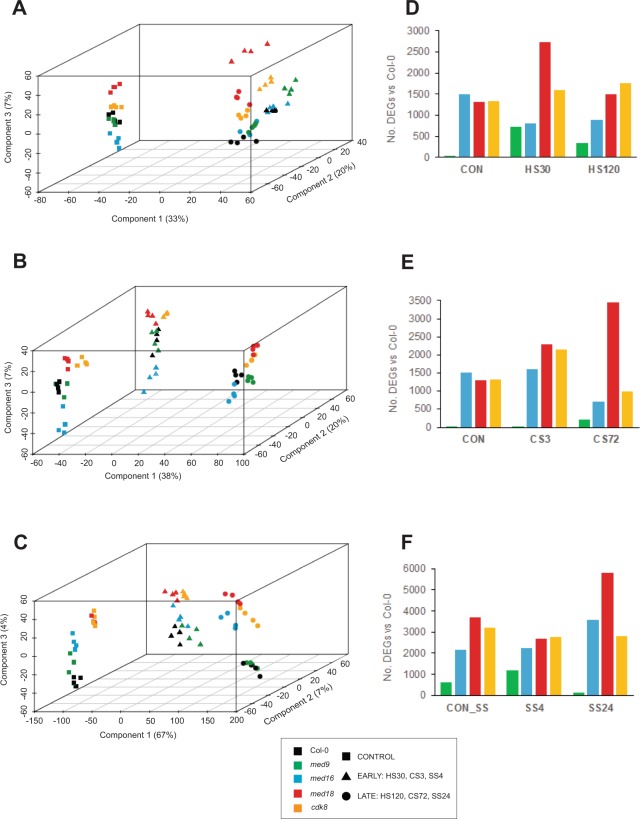
Global comparison of *Arabidopsis* Col-0 and Mediator mutant transcriptomes in control and abiotic stress conditions. Each data point represents the entire transcriptome for each sample, and are arranged in the first, second and third dimensions according to the components of a principal component analysis (PCA; shown on the x, z and y axes, respectively). Each transcriptome from the (**A**) heat, (**B**) cold and (**C**) salt stress experiments is shown as one data point. Transcriptomes from plants in control conditions are shown as squares; those from early stress time-points (HS30, CS3 and SS4) are shown as triangles; and those from late time-points (HS120, CS72 and SS24) are shown as circles. Col-0 wild type: black; *med9*: green; *med16*: cyan; *med18*: red; *cdk8*: orange. The contribution of each component to the total variation between samples is shown as a percentage. (D-F) Total numbers of significant differentially expressed genes (DEGs; *p*_adj_ ≤ 0.01 and log_2_ fold-change ≥ ± 0.5) between Col-0 and Mediator mutant transcriptomes in each stress.

To assess global differences between Col-0 and mutant transcriptomes, we performed a principal component analysis (PCA). The data were normalized using a variance-stabilizing transformation (VST) and filtered for lowly-expressed genes, yielding datasets of 24,450, 24,194 and 25,914 transcripts for the heat, cold, and salt stress experiments, respectively. For each stress, the PCA revealed large-scale grouping of Col-0 and mutant transcriptomes into three clusters corresponding to the three time-points in each experiment, with transcriptomes in the LATE time-points clustering furthest from those in CON (Fig. 2A–C). The variation in the first two PCA components was most likely attributable to time and accounted for 33%, 38%, and 67% of the variation in the first principal components (Fig. 2A–C; x-axes), and 20%, 20% and 7% in the second components (Fig. 2A–C; z-axes), for heat, cold and salt stress, respectively.

The third component revealed separation by genotype, accounting for 4–7% of the total variation in each experiment (Fig. 2A–C; y-axes). In transcriptomes from the CON time-point, Col-0 and *med9* clustered together (Fig. 2A,B); however, in hydroponic conditions (CON_SS) we observed difference between these transcriptomes (Fig. 2C). In CON, we also noticed that the transcriptomes of *med16*, *med18* and *cdk8* diverged from Col-0: *med18* and *cdk8* clustered together, while transcriptomes from *med16* formed distinct clusters in the opposite direction (Fig. 2A,B). This suggests antagonistic effects of tail subunit deletion on Mediator function and gene expression, relative to head and CKM subunit deletions. Indeed, of the 798 genes significantly downregulated in *med16* in CON (see below), 144 (18%) were significantly upregulated in *cdk8* at the same time-point, and 120 (15%) in *med18* (with 75 shared between *med18* and *cdk8*) (Supplemental Table S1).

To quantify differences between Col-0 and the mutants’ transcriptomes observed in the PCA, we calculated total numbers of differentially-expressed genes (DEGs) in the mutants compared with Col-0 at each time-point (see Methods and^41^). The total number of DEGs in each mutant recapitulated their differences from Col-0 observed in the PCAs (Fig. 2D–F; Supplemental Table S1).

### The transcriptional response of Col-0 to abiotic stresses includes common and stress-specific functional gene categories

**Figure 3:**
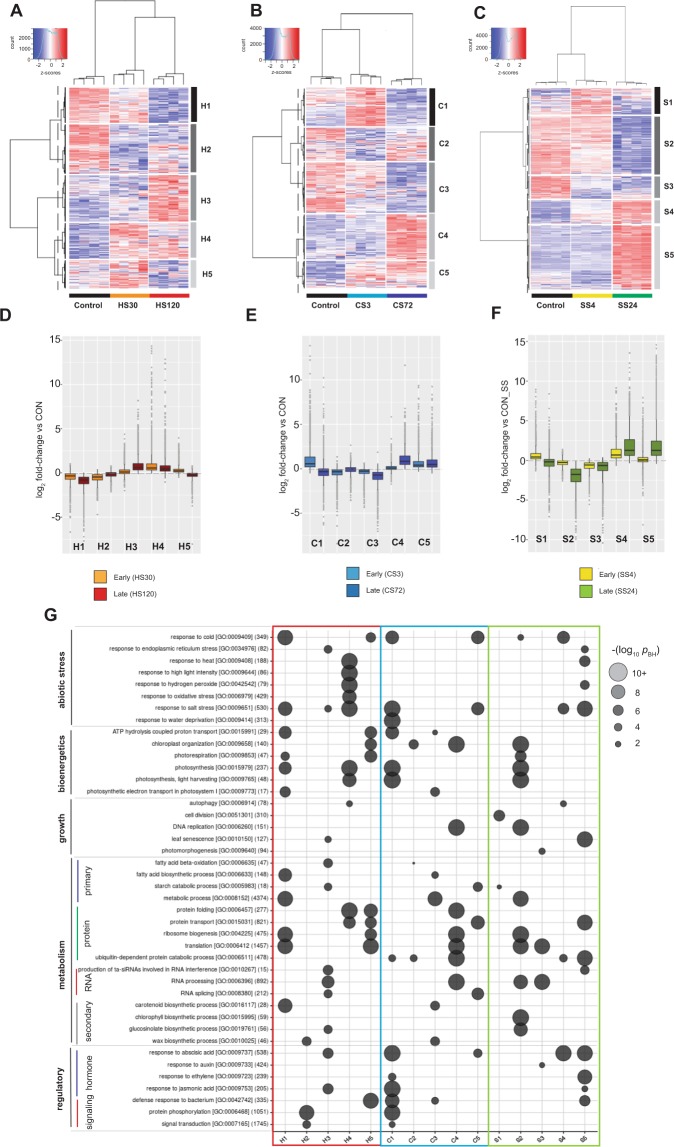
The transcriptional response of *Arabidopsis* Col-0 plants to abiotic stress. The transcriptional response of Col-0 wild type to (**A**,**D**) heat, (**B**,**E**) cold or (**C**,**F**) salt stress is shown. (**A–C**) Hierarchical clustering of co-expressed genes, differentially regulated in response to (A) heat-, (**B**) cold-or (**C**) salt stress, either in control conditions or early or late phase of stress. VST-normalised data for around 15,000 filtered transcripts are displayed as z-scores, and cluster dendrograms are shown with a dashed line indicating divisions between 5 co-expressed clusters (H1-5, C1-5 or S1-5 for heat clusters 1–5, cold clusters 1–5, or salt clusters 1–5, respectively). (**D–F**) Summary boxplots indicating log_2_ fold-change (relative to expression in Control conditions) for all transcripts in each of the 5 co-expressed gene clusters in (**D**) heat, (**E**) cold or (**F**) salt stress. Boxes indicate the first quartile, the median, and the third quartile. The whiskers indicate the range of no more than 1.5 times the interquartile, and outliers are individually marked. Fold-change data from both the early and late stress time-points in each experiment are shown. (**G**) Gene ontology (GO) enrichment analysis of each co-expressed cluster in stress experiments. The size of the circle and colour intensity indicates the significance (–log *p*-value (Benjamini-Hochberg adjusted)) of the functional enrichment for each category. The total number of genes from each GO category in *Arabidopsis thaliana*, which were present in our detected population, is shown in parentheses after their GO consortium IDs. Some functional redundant functional categories were excluded for clarity.

Next, we analyzed the transcriptional response of Col-0 to each stress. We performed hierarchical clustering on the VST-normalized data and generated heatmaps, with the resulting normalized gene expression for all replicates displayed as z-scores (Fig. 3A–C). For each stress we identified five clusters of 1,200–4,800 co-expressed transcripts displaying a similar temporal response (Supplemental Table S2). GO analysis of these clusters revealed both similar and unique responses. In the EARLY phases of all stress experiments, we detected upregulation of clusters (H4, H5, C1, C5, S1, and S4) enriched in genes required for responses to abiotic stress. In heat, this included classical heat-responsive genes encoding chaperones, and proteins involved in photosynthesis and photorespiration (Fig. 3A,D,G)^42^. In cold, we observed upregulation of transcripts for ABA and JA responses, drought, salt and cold stress, RNA splicing, photosynthesis, protein transport and starch catabolism (Fig. 3B,E,G). In salt stress, ABA, salt and cold-responsive transcripts as well as those for starch catabolism, cell division, response to endoplasmic reticulum (ER) stress and ubiquitin-dependent protein catabolism were upregulated (Fig. 3C,F,G). Transcripts for autophagy genes were also upregulated in both the heat and salt stress experiments.

In each stress we detected a large cluster of downregulated transcripts in both EARLY and LATE phases. In heat, H1 contained downregulated transcripts for ribosome biogenesis and translation, fatty acid and carotenoid biosynthesis, and photosynthesis. The equivalent cluster (C3) in cold stress also included transcripts for central and secondary metabolism and photosynthesis, and transcripts for ATP hydrolysis-coupled proton transport were downregulated in both heat and cold (H1, C1 and C3). In salt stress, we detected downregulated transcripts at both early and late time-points for RNA processing, translation, photomorphogenesis and auxin response (S3).

Finally, we observed the largest clusters in the LATE phases of each stress. In heat, transcripts for ER and salt stress, RNA metabolism, fatty acid and glucosinolate metabolism and ABA and JA signaling were upregulated (H3). In the LATE phase of cold, transcripts involved in protein metabolism, DNA replication, RNA processing and chloroplast organisation were upregulated (S4). Upregulation of ubiquitin-dependent protein catabolism, heat and salt stress-response transcripts was also observed in the LATE phase of salt stress (S5), as were transcripts for leaf senescence, ta-siRNA-mediated gene silencing and response to ABA, H_2_O_2_ and ethylene. As in other stresses, we detected a large set of downregulated transcripts at the late time-point in salt for translation and ribosome biogenesis, RNA and secondary metabolism, photosynthesis and photorespiration (S2).

### Identification of common stress-response regulons

**Figure 4:**
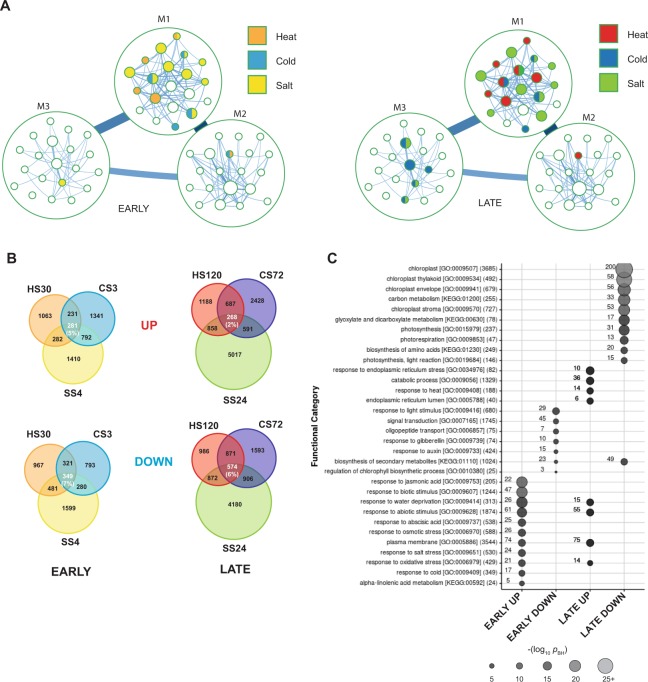
Common abiotic stress regulons in *Arabidopsis* Col-0. (**A**) Gene co-expression network visualizing the 20 largest modules at the first and second levels of organisation. Modules are colored by significantly enriched gene-sets according to a hypergeometric test. For modules with more than one color, more than one gene-set was significant. (**B**) Overlaps between sets of significantly upregulated (UP) or downregulated (DOWN) transcripts in Col-0 wild type at early (EARLY stress regulons) and late time-points (LATE stress regulons). (**C**) Gene ontology (GO) and KEGG pathway enrichment analysis of the EARLY and LATE UP and DOWN stress regulons. The size of the circle and color intensity indicates the significance (−log *p*-value (Benjamini-Hochberg adjusted)) of the enrichment for each functional category. Numbers beside each circle indicate the number of genes attributed to each category that are present in each regulon. The total number of genes from each GO category in *Arabidopsis thaliana*, which were present in our detected population, is shown in parentheses after their GO consortium or KEGG pathway ID. Some redundant functional categories were excluded for clarity.

We next defined the set of DEGs in response to each abiotic stress and time-point, calculated in comparison to the expression level in control conditions (CON or CON_SS; Supplemental Table S3). To visualize the stress-related DEGs and identify patterns of co-regulation between stresses, we created a partitioned gene co-expression network, where similarly regulated genes are grouped into modules (Fig. 4A; Supplemental Table S4). We found substantial partitions at the highest two levels of organisation, while at the third level almost all modules were composed of single genes. At the first level we identified three large modules (M1, M2 and M3) containing 9,805, 4,116 and 1,589 genes each, and several small modules containing two genes or less. At the second level of organization, we observed that the three major first-level modules were subdivided into 578 smaller modules (M1:1-M1:377, M2:1-M2:123, M3:1-M3:78). The majority of the second-level modules were small, and the 20 largest modules contained nearly two-thirds of the genes present in the first-level modules. We analysed the enrichment of stress-related sets of genes among the identified modules and found that the majority of the early stress genes were found in the top-level M1 module (Fig. 4A; Supplemental Table S5). In the second-level modules, early heat stress genes were enriched in a few distinct modules (5) but these showed very little overlap (and therefore possible co-regulation) with that seen for the other two stress conditions. In contrast, cold stress genes were enriched in five second-level modules which often overlapped with modules containing salt-stress genes (3/5), suggesting a possible co-regulation of genes required for response to these two abiotic stresses. Salt stress genes generally displayed a more dispersed pattern and were enriched in the largest number of modules (11), indicating that salt affects a more general set of processes with distinct regulatory patterns. A similar pattern was detected for the late-responsive genes: of the identified early stress gene-containing network modules, 5/5 heat modules, 3/5 cold modules and 8/11 salt modules were again enriched for the same stress. Interestingly, more modules were enriched overall in the late response, especially in module M3, indicating that the expression of additional gene networks had been activated or suppressed by the late stage of stress.

To focus on key common stress-response genes and reduce the complexity of our dataset, we identified the overlapping set of DEGs which were up- or downregulated in all three stresses at the EARLY and LATE time-points (Fig. 4B). Of the 1,857, 2,645 and 2,765 transcripts upregulated in Col-0 at HS30, CS3 and SS4 (Supplemental Table S3), we identified a common regulon of 281 genes (EARLY UP, ~5% of the total) whose expression was upregulated in all stresses (Fig. 4B, upper panel). Similarly, of the 2,118, 1,743 and 2,709 downregulated DEGs identified at HS30, CS3 and SS4, respectively, we identified a common regulon of 349 genes (EARLY DOWN, ~7% of the total) (Fig. 4B).

In the LATE phase of stress, we identified 3,001, 3,974 and 6,734 upregulated transcripts at HS120, CS72 and SS24, respectively, and 3,303, 3,944 and 6,532 transcripts which were downregulated. We detected 268 DEGs which were upregulated in the LATE phases, forming a LATE UP regulon (2% of the total) and 574 DEGs common for all stresses forming a LATE DOWN regulon (6% of the total). These will onwards be referred to as the four common stress regulons; genes in each regulon and their expression fold-changes in response to each stress are shown in Supplemental Table S6.

GO analysis revealed that the EARLY UP was enriched in abiotic stress response genes and many plasma membrane-localized signaling and hormone metabolism components (Fig. 4C). In the EARLY DOWN, transcripts for components of signal transduction, oligopeptide transport, biosynthesis of secondary metabolites and response to light, auxin and GA were downregulated. The EARLY regulons also include a high proportion of genes for TFs: 44/281 upregulated (16%) and 43/349 (12%) downregulated, which is much higher than the background in *Arabidopsis* according to PlantTFDB (2,086 of 25,914 detected transcripts in our background ≈ 8%). In the LATE regulons, transcripts for 55 abiotic stress-response genes were upregulated, including 21 already upregulated in the EARLY regulon (Supplemental Table S6). A number of transcripts involved in response to ER stress were also upregulated in the LATE phase, consistent with the accumulation of misfolded proteins recently identified as a common factor in abiotic and biotic stresses^2,43^. The largest numbers of common stress-responsive transcripts were downregulated in the LATE phases (Fig. 4B). GO analysis revealed that they were enriched in transcripts encoding chloroplast-localized proteins, including photosynthesis and carbon metabolism, photorespiration, glyoxylate and dicarboxylate metabolism and biosynthesis of secondary metabolites and amino acids.

These results are consistent with the concept of a fast transcriptional acclimation response, including induction of essential stress-response transcripts and altered levels of regulatory, transcriptional and signaling transcripts, followed by maintained stress response and downregulation of transcripts for primary and secondary metabolism and bioenergetics as plants adapt to the suboptimal conditions^44^.

### Enrichment of TF binding sites in promoters of DEGs in the stress regulons indicates sequential binding of specific TF-families

**Figure 5:**
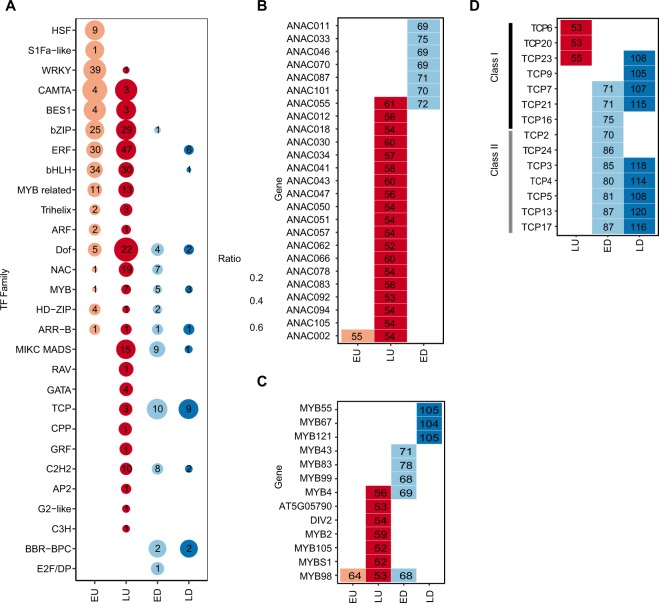
Enriched transcription factor binding sites in the four common abiotic stress regulons. Genes from the four stress regulons EARLY UP (EU), LATE UP (LU), EARLY DOWN (ED), and LATE DOWN (LD) were analyzed using TF2Network^45^. Significantly enriched transcription factor (TF) binding sites were summarized to the level of TF-families (**A**) (An extended list of all significantly enriched TFs can be found in Supplemental Fig. S4). Numbers within circles indicate the number of significant family members and the size of the circle indicates the proportion of the family that is significantly enriched. All members of the TF-families NAC and MYB that are significantly enriched in at least one of the four stress regulons are found in (**B**,**C**), while Class I and II members of the TCP family are indicated in (**D**). Numbers within rectangles indicate the number of target genes enriched for the respective TF.

Plant transcriptional responses to abiotic stress require a network of TFs^2^. We used TF2Network^45^ to identify enriched TF binding sites (TFBS) in the promoters of DEGs for each of the four common stress regulons. In total, 341 TFBS were enriched in promoters of the four regulons relative to the entire genome (Supplemental Fig. S4). Some TFBS were found in more than one regulon, but the majority (196 sites) was unique to one. We grouped the TFBS into families according to the Plant Transcription Factor Database 4.0^46^ and sorted them according to their abundance in each regulon (Fig. 5A). We observed a wave-like sequence in the UP regulons; one group was exclusively enriched in the EARLY UP, followed by a second group enriched both in the EARLY and LATE UP. Finally, a third group of TF-families was exclusively enriched in the LATE UP.

The first group was enriched for the HEAT SHOCK FACTOR (HSF), WRKY and S1Fa-like families. HSFs are major regulators of plant heat response, but have also been implicated in activation of target genes for cold, osmotic and salt stress^47^. TFBS for nearly half (9 of 21) of all HSFs were enriched in the EARLY UP (Supplemental Fig. S4). The WRKY family comprises 72 members and more than half (39 of 72) of these were enriched in the EARLY UP. Finally, we also observed enrichment for one (AT3G09735) of three members of the S1Fa-like family in the EARLY UP regulon.

The second group was enriched for families that share structural motifs, including the basic helix-loop-helix (bHLH), basic region/leucine zipper motif (bZIP), Myb-related, Trihelix and HD-ZIP families (Fig. 5A; Supplemental Fig. S4). It also contained a set of functionally-related TF families, including a large group (55 of 121 members) representing the ETHYLENE RESPONSE FACTOR (ERF) family, including the DREBs, which are key abiotic stress-response regulators^48^, the BES1 family (4 of 8) which are involved in activation of BR-induced genes^49^, and the CALMODULIN-BINDING TRANSCRIPTIONAL ACTIVATOR (CAMTA) family (4 of 6), which functions in both activation and repression of abiotic and biotic stress-response genes^50^.

The third group included TF-families which were preferentially enriched in the LATE UP, including the NO APICAL MERISTEM/*ARABIDOPSIS* TRANSCRIPTION INITITATION FACTOR/CUP-SHAPED COTYLEDON (NAC) family (19 of 110) which recently has been shown to have key functions in abiotic and biotic stress responses^51,52^, and the DNA-BINDING WITH ONE FINGER (DOF) family (22 of 36), which participates in regulation of seed development, carbohydrate metabolism, biotic stress and auxin/GA responses^53^. They have also been shown to participate in abiotic stress responses^54^. The third group also included the structurally-related MIKC-MADS (15 of 41), C2H2 (10 of 100), GATA (4 of 30), TCP (3 of 24) and Myb (7 of 144) families, and some families that showed enrichment of only one TF.

The EARLY and LATE DOWN were enriched for fewer TFBS. The DOWN regulons also showed a wave-like temporal sequence. In the first group, we were surprised to again find enrichment of NAC-family proteins, as in the LATE UP (cf. columns 2 and 3 in Figs. 5A and S5). A more detailed analysis revealed that a distinct set of individual NAC-proteins was enriched in the EARLY DOWN compared the LATE UP, with the exception of ANAC055 (Fig. 5B). This suggests a separation of the NAC-family proteins into two subfamilies with opposite functions. We also found enrichment of TFs belonging to the bZIP, HD-ZIP, MIKC-MADS, C2H2 and E2F/DP families in the EARLY DOWN.

The second group of TFs contained the Myb family which was also enriched in the EARLY and LATE UP. Like the NAC family, we found that each regulon was enriched for distinct individual Myb-TFs (Fig. 5C). One interesting exception was MYB98, which was enriched in the EARLY and LATE UP and the EARLY DOWN, and which is known to be upregulated in oxidative stress response induced by methyl viologen treatment^55^. We also identified the TCP family as enriched in both EARLY and LATE DOWN as well as in the LATE UP. Like the NACs, we found a distinct set of TCPs enriched in the LATE UP compared to the EARLY and LATE DOWN (Fig. 5D). All TCPs in the LATE UP belong to the Class I subfamily, while most TCPs in the EARLY and LATE DOWN belong to the Class II subfamily^56^. In addition, the second group included members of the DOF, ARR-B, MYB and BBR-BPC families.

The last group of TFs, which were enriched predominantly in the LATE DOWN promoters, comprised one bHLH and six ERF family members. Several ERFs were also enriched in the EARLY- and LATE UP, but we noticed that both ERFs that were uniquely enriched in the LATE DOWN belong to the DREB subfamily A-5 (DEAR3 and DEAR5; Supplemental Fig. S4). These proteins likely contain an EAR motif which promotes transcriptional repression^57,58^.

### Effects of mediator subunit mutations on the early response to stress

**Figure 6:**
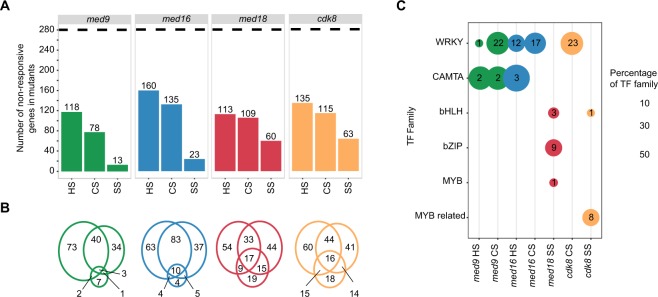
The EARLY UP common abiotic stress regulon in Mediator mutants. (**A**) Bar graphs of numbers of genes out of the EARLY UP regulon which were not significantly upregulated (non-responsive) in the Mediator mutants in early heat (HS), cold (CS) and salt stress (SS). The dotted line indicates the total number of genes in the EARLY UP regulon (281). (**B**) Venn diagram showing the overlap between stresses for each mutant. In each Venn diagram the left, right and lower circles correspond to HS, CS and SS, respectively. (**C**) Enrichment analysis of transcription factor (TF) binding sites in the non-responsive genes in the mutants as compared to the full group of 281 genes, summarized to the level of TF-families (An extended list of all significantly enriched transcription factors can be found in Supplemental Fig. S5). Numbers within the circles indicate the number of significant family members and the size of the circle indicates the proportion of the family that was significantly enriched.

To identify requirements for Mediator subunits in stress-responsive gene expression, we compared expression of genes in the EARLY UP in Col-0 with their expression at the early time point after heat, cold and salt stress in each mutant. We identified stress-responsive transcripts which, in the Mediator mutants, did not respond during early stress; hereafter, these will be referred to as ‘non-responsive’ genes. Of the 281 DEGs in the EARLY UP regulon, 118, 78 and 13 were non-responsive in *med9* in the heat, cold and salt stress experiments, respectively (Fig. 6A; Supplemental Table S7). The corresponding numbers for the other mutants were 160, 135, and 23 (*med16*), 113, 109, and 60 (*med18*) and 135, 115 and 63 (*cdk8*). This indicates that all four subunits are involved in the early induction of target genes for thermal stress. In contrast, *med18* and *cdk8* displayed more dysregulated early salt responses. We found considerable overlap of non-responsive genes for thermal stresses primarily in *med16* (Fig. 6B), indicating that TFs involved in both heat and cold stress responses might interact with MED16. The importance of MED16 for cold response in *Arabidopsis* is well-documented^27^, but it has not previously been implicated in heat response. However, this function of MED16 may be evolutionarily conserved, since MED16 is involved in heat response in *S. cerevisiae*^59,60^.

### MED9, MED16 and CDK8 are required for WRKY-regulated early thermal stress-responsive gene expression

To identify TFs that depend on specific Mediator subunits for early stress-specific gene induction, we analyzed the lists of non-responsive genes to identify enrichment of TFBS in their promoters relative to those in the EARLY UP. For the 12 possible combinations, we identified significant enrichment of 53 TFs in seven of the conditions/mutants (Fig. 6C; Supplemental Fig. S5). We found similar patterns of enriched TF-families in the list of non-responsive genes in *med9*, *med16* and *cdk8* in thermal stress response. TFBS for 22, 17 and 23 members of the WRKY family were enriched among non-responsive genes in *med9*, *med16* and *cdk8*, respectively, in early cold. We recently showed that a *cdk8* mutant was unable to respond properly to cold^26,39^. Our results suggest that WRKYs are involved in this process, consistent with reported interactions with CDK8^61^ and that overexpression of WRKYs results in increased cold tolerance^62^. AtMED9 was recently shown to interact with MED4, MED21 and MED31 *in planta* and to display transactivation activity in yeast^63^. Combined with our results identifying a connection between MED9 and WRKYs, these data provide the first descriptions of MED9 function in plants. Finally, we observed enrichment of WRKY-family consensus sites in promoters of the non-responsive genes in *med16* in the early response to heat. This strengthens the above-mentioned results indicating that MED16 is important for the heat stress response and suggests that WRKYs are involved in this process.

### MED9 and MED16 are involved in induction of target genes by CAMTA TFs in the early thermal stress response

A second TF family which functioned improperly in specific Mediator mutants in the early stress response was the CAMTAs. Relative to the EARLY UP in Col-0, CAMTA TFBS were enriched in promoters of non-responsive genes in *med16* in heat and in *med9* in heat and cold (Fig. 6C; Supplemental Fig. S5). CAMTA1–3 were shown to participate in cold tolerance by cooperatively inducing CBF target genes and repressing SA biosynthesis, and a *camta1/3* double mutant displayed impaired freezing tolerance^64,65^. MED16 has a known function in SA and cold-response signaling and a recent report showed that mutations in either *CDK8* or *MED12* can partially suppress the SA hyper-accumulation phenotype of a *camta1/2/3* triple mutant^66,67^.

### Non-responsive EARLY UP genes connected to thermal stress are enriched in network module M1:7

Returning to the gene co-expression network, we observed that 126 of the 281 genes from the EARLY UP regulon gene-set were significantly enriched in three modules (M1:7, M1:3, M2:6), while the remaining 155 genes were spread out over the network (Supplemental Table S5; Supplemental Fig. S6). Using a hypergeometric test, we determined if the non-responsive genes in the mutants co-occurred significantly in any of these three modules (Table 1). Interestingly, the only module enriched for non-responsive genes was M1:7. This module was enriched for non-responsive genes in *cdk8* during cold stress, *med9* in heat and cold stress, and *med16* in heat and cold stress. Module M1:7 contains of 390 genes, and a TFBS analysis indicated that these genes were enriched for CAMTA (48% of targets) and WRKY-family (30% of targets) TF binding sites (Supplemental Table S8). Co-localisation of these non-responsive genes within a module containing co-expressed genes also enriched for CAMTA and WRKY TFBS provides support for the biological relevance of the results presented above.

**Table 1:** Modules significant for non-responding genes.

	Gene-set	M1:3	M1:7	M2:6	Total genes
	Genes in module	714	390	185	NA
	EARLY UP	44	56	26	281
*med9*	CS	0 (p = 1)	29 (p = 1.9E-04)	11 (p = 1)	78
	HS	17 (p = 1)	42 (p = 1.9E-07)	10 (p = 1)	118
	SS	1 (p = 1)	0 (p = 1)	1 (p = 1)	13
*med16*	CS	1 (p = 1)	46 (p = 1.0E-05)	16 (p = 1)	152
	HS	22 (p = 1)	53 (p = 1.2E-11)	13 (p = 1)	160
	SS	4 (p = 1)	1 (p = 1)	5 (p = 0.57)	23
*med18*	CS	0 (p = 1)	20 (p = 1)	13 (p = 1)	116
	HS	22 (p = 1)	22 (p = 1)	16 (p = 0.29)	113
	SS	5 (p = 1)	9 (p = 1)	6 (p = 1)	60
*cdk8*	CS	0 (p = 1)	34 (p = 0.01)	12 (p = 1)	115
	HS	24 (p = 1)	26 (p = 1)	17 (p = 0.91)	135
	SS	5 (p = 1)	7 (p = 1)	10 (p = 0.67)	63

### MED16 is required for WRKY, HSF and CAMTA TF-dependent repression of target genes for the early cold stress response in non-stress cells

Two mechanisms could explain non-responsiveness of a target stress gene in a mutant. Either, the mutant expresses the gene at the same low level as Col-0 before stress but cannot induce its expression; or, the target gene is already de-repressed in the mutant relative to Col-0 prior to stress but is not further induced upon stress. In the first case, non-responsiveness indicates the inability of a stress-response activator to interact with Mediator lacking a specific subunit. In the second case, it suggests that a repressor which is active in Col-0 prior to stress is unable to interact with mutant Mediator.

**Figure 7:**
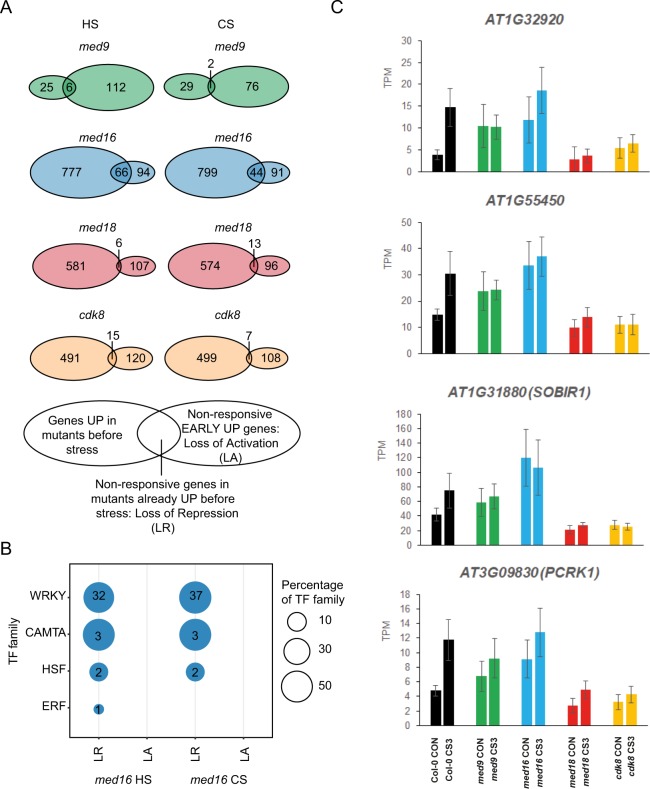
Loss of repression among non-responsive genes in the EARLY UP common abiotic stress regulon. (**A**) Venn diagram showing the overlap between non-responsive genes in mutants from the EARLY UP stress regulon and the genes already significantly upregulated prior to stress in the mutants, compared to Col-0. The overlap between the two circles represents genes that are non-responsive in mutants due to a loss of repression/increased expression prior to stress exposure. (**B**) Transcription factor (TF) binding site enrichment analysis was repeated for *med16* after non-responsive genes were divided into loss of repression (LR) and loss of activation (LA) according to the Venn diagram. Results were summarized at the level of TF-families. (An extended list of all significantly enriched TFs can be found in Supplemental Fig. S7). (**C**) Expression levels of representative genes (displaying LR in the *med16* mutant prior to stress). Data shown are the average transcripts per million (TPM) ± SD in control conditions (CON) or early cold stress (CS3).

To distinguish between these mechanisms, we identified the overlap between genes that are upregulated prior to stress, and the non-responsive genes at the early time-points of heat and cold in each Mediator mutant (Fig. 7A). These overlaps represent genes that fail to respond properly due to loss of repression (LR) in mutants already prior to stress. In contrast, genes that are non-responsive due to loss of activation (LA) are found in the non-overlapping rightmost sectors in each Venn diagram. We found that *med16* shows a unique overall pattern relative to the other mutants, displaying a much larger fraction of the genes that fail to be induced by stress due to LR prior to stress.

We next analyzed the genes in each of the two types of non-responsive genes for each mutant and each stress, to see if their promoters were enriched for any TFBS relative to all promoters in the EARLY UP. For both heat and cold, we found enrichment of TFBS only among the 66 (HS) and 44 (CS) genes displaying LR in *med16*. In both stresses, we observed enrichment of TFBS of the WRKY, CAMTA and HSF TF-families, and one ERF protein (ERF34) (Fig. 7B; Supplemental Fig. S7). In contrast, the 94 (heat) and 91 (cold) EARLY UP genes displaying LA in *med16* showed no enrichment of TFBS (Fig. 7B).

These results, combined with those in Figs. 6 and S7, suggest that *med9*, *med16* and *cdk8* show a similar inability to induce transcription of key stress genes that are regulated by the WRKY, CAMTA (in *med9* and *med16*) and HSF TF-families (in *med16*), but that the underlying mechanism is different. Prior to stress, MED16 appears to be required for repression at these loci, while MED9 and/or CDK8 are involved in activation of thermal stress target genes. For example, *AT1G32920*, *AT1G55450*, *AT2G31880* (*SOBIR1*) and *AT3G09830* (*PCRK1*) were all induced in Col-0 in the early phases of cold stress, but displayed LR in *med16* prior to stress, though their final expression levels were similar (Fig. 7C). In contrast, in *med9*, *med18* and *cdk8*, these genes displayed similar levels of expression as in Col-0 prior to stress, but their induction in cold was impaired. These results reinforce our PCA results (c.f. Fig. 2A–C) which also indicated that MED16 has an antagonistic function in gene expression compared to head- and CKM-module subunits.

### CDK8 is required for function of circadian clock TFs, and MED18 for function of bZIP, bHLH, and Myb TFs, in the early salt stress response

The results above describe early cold and heat responses, where most effects relative to Col-0 were observed in *med9*, *med16* and *cdk8* and their inability to respond properly to the WRKY, CAMTA and HSF TF-families. However, we also noticed enrichment of nine TFs which function improperly in *cdk8* in salt response (Fig. 6C; Supplemental Fig. S5). Interestingly, eight of these (LHY, CCA1, and RVE1, 3, 4, 5, 6, 8) belong to a subgroup of 11 Myb-related TFs primarily associated with circadian clock functions^68^. However, LHY, CCA1, and RVE4/8 have also been identified as key regulators of abiotic stress responses^69,70^.

The *med18* mutant displayed the highest level of transcriptional dysregulation (Fig. 2). Analysis of EARLY UP in *med18* identified 113, 116 and 60 non-responsive genes at HS30, CS3 and SS4, respectively (Fig. 6). Even so, we only found enrichment of TFBS in promoters of the non-responsive genes in early salt stress (Fig. 6C; Supplemental Fig. S5). These corresponded to the bZIP, bHLH and Myb families, several of which have been implicated in abiotic stress. In particular, we found enrichment of two bHLH family TFs (PIF1, PIF3) which are central regulators of light signaling^71^. We also found enrichment of the C/S_1_ group of bZIP TFs, such as bZIP11 (ATB2), bZIP53 and bZIP63, which form heterodimers and activate low-energy signaling networks in abiotic stress responses^72,73^.

### Promoters of genes in the EARLY DOWN regulon are enriched in binding sites for specific CDK8-dependent TF families

We next analyzed the 349 genes in the EARLY DOWN to identify non-responsive target genes in the transcriptomes of each mutant and stress condition. We found that 78, 91 and 26 of the 349 DEGs in the EARLY DOWN were non-responsive in *med9* in heat, cold and salt stress, respectively (Supplemental Fig. S8A). The corresponding numbers for the other mutants were 72, 165, and 49 (*med16*); 75, 142, and 92 (*med18*); and 87, 132 and 76 (*cdk8*). As in the EARLY UP, this indicates that all mutants have roles in repression of target genes for early thermal stress response, but in this case the largest effects observed were in response to cold in *med16*, *med18* and *cdk8*. The strongest effect was observed in cold in *med16*, in which almost 50% of the target genes of the EARLY DOWN were dysregulated, again supporting the reported cold-sensitive phenotype of *med16*^27^.

As for the EARLY UP, for non-responsive genes in each Mediator mutant and stress, we identified TFBS which were enriched relative to the EARLY DOWN. We found far fewer TFBS in this analysis, compared to the EARLY UP (Figs. 6C and S6). The only significant enrichment was in *cdk8*, where promoters of genes that were non-responsive to salt stress were enriched for TCP2 and MYB98 TFBS (Supplemental Fig. S8B). These genes were primarily associated with light signaling, including *EPS1, ERD9* and *CYP711A1*, and carbon metabolism, such as *DIN9*, *BGAL8*, and *RPI2*. While their expression was downregulated in Col-0 at SS4, they were less responsive in *cdk8* (Supplemental Fig. S8C).

## Discussion

Here we used a Col-0 wild-type line and four Mediator mutant lines to study Mediator function in expression of stress-responsive genes during heat, cold and salt stress. Globally, we observed the most variation when comparing time-points, rather than between lines/mutants, indicating that all mutants generally responded to each stress in a similar way as Col-0. However, we also identified stress-specific differences between lines.

In all stresses, we identified clusters of co-expressed transcripts displaying similar temporal responses in Col-0. Focusing on genes that were commonly regulated in all stresses in Col-0, in the EARLY phases of stress, we found that a strong transcriptional response is initiated to prevent or ameliorate irreversible damage. This includes upregulation of genes encoding chaperones and ROS-scavenging enzymes during heat stress, ABA/water deprivation and cold-responsive genes in cold stress, and salt/ABA-responsive ionic transporters in salt stress, consistent with the prevailing view^2^. Starch catabolism was also upregulated in early cold and salt stress, indicative of the energetic requirements of stress acclimation. In the LATE phases, we detected downregulation of translation, photosynthesis and primary and secondary metabolism genes in all stresses, indicating large-scale transcriptional reprogramming to counteract the detrimental effects of abiotic stress on photosynthesis, growth and development^74^. In line with this, we recently suggested that Mediator may be a key component in this metabolic switch between growth/development and stress response^75^.

The UP and DOWN regulons both displayed an interesting wave-like, temporal sequence of TFBS enrichment indicating sets of TF-families which control the early and late stress responses. Early stress involved recruitment of HSFs and WRKYs, which are important for response to specific stresses^76,77^, but our results suggest more general functions in abiotic stress. This was followed by a second wave comprising the ERF, bZIP, BES1, CAMTA, bHLH and Myb-related families. These are also important for the early, general stress response but are also important for regulation of target genes of the late responses. The last wave included members of several TF-families, including DOF, NAC, MYB and MIKC-MADS.

Target genes in the DOWN regulons also showed a wave-like TFBS enrichment sequence. The EARLY DOWN promoters were enriched for the NAC, C2H2, HD-ZIP and MIKC-MADS-families. Notably, while NAC-family proteins were enriched in promoters of both the LATE UP and EARLY DOWN, we found that the different individual NAC-proteins were identified in the LATE UP compared with the EARLY DOWN, suggesting that members of this family have antagonistic functions. The MYB-family was also enriched in all four regulons and showed almost complete separation of individual MYBs into each regulon. The most prominent TF-families in the second wave were the DOFs, MYBs, and TCPs. Like the NACs, a different set of TCPs were enriched in the LATE UP compared with the EARLY and LATE DOWN. All TCPs in the LATE UP belong to the Class I subfamily, suggesting that the TCP family also includes proteins with opposite functions in abiotic stress. Antagonistic effects of Class I and Class II TCPs in regulation of specific processes have been reported previously^56^. Finally, only the ERF and bHLH families were enriched uniquely in the LATE DOWN. The individual TFs that we identify here have previously been identified in specific types of stress responses. However, our results extend these findings by assigning several of the TF-families to a more general function in response to different types of stress, and by revealing their temporal and combinatorial organisation.

In early stress, the Mediator mutants all displayed transcriptional dysregulation, with some distinctions: thermal stress response was particularly dysregulated in *med9* and *med16* and salt stress response in *med18* and *cdk8*. Many key genes regulated by WRKY and CAMTA family TFs required MED9 and MED16 for the thermal response and CDK8 in cold. Similarly, a module in our gene co-expression network which was enriched for non-responsive EARLY UP genes in these mutants was also enriched for genes with CAMTA and WRKY TFBS, reinforcing the biological relevance of the interaction between these TFs and Mediator in abiotic stress response. An interesting finding was that MED16 was specifically required for repression of CAMTA, WRKY and HSF target genes prior to stress. A co-repressor function for *Arabidopsis* MED16 has recently been described, as it interacts with the transcriptional repressor DEL1^78^. In yeast, Med16 is a co-repressor of a key heat-stress gene prior to stress^60^. De-repression of stress-response genes prior to stress might indicate pre-adaption and may explain why some mutants display increased resistance to certain stresses. Furthermore, we observed that deletion of different Mediator subunits resulted in different effects on gene expression from the same loci: MED16 deletion resulted in a loss of repression prior to stress, while *med9* and *cdk8* displayed loss of activation in response to stress. PCA indicated antagonistic effects on global gene expression in *med16* compared to *med18* and *cdk8*, which clustered together. This suggests that different modules operate by distinct mechanisms, possibly including phosphorylation of Mediator subunits or cognate TFs by CDK8. This is consistent with a recently detected genetic interaction between *Arabidopsis* CDK8 and another tail module subunit (MED5)^79^. Furthermore, in yeast and human cells, MED18 has been shown to function in the same regulatory pathways as their respective CDK8 proteins, consistent with our results^80,81^.

The WRKY and CAMTA TF-families have been implicated in several abiotic stress response signaling pathways, including JA/ET, SA, and ABA^82,83^. Furthermore, several reports suggest that Mediator is involved in crosstalk between JA/ET and ABA signaling components, especially via the MED25 subunit, which has been shown to interact with MYC2 and the ABA-associated TF ABI5^84,85^. MED25 was recently shown to directly link the JA receptor CORONATINE INSENSITIVE 1 (COI1) with promoters of MYC2 target genes, leading to degradation of JAZ-domain transcriptional repressors, HAC1-dependent H3K9 acetylation, and activation of JA-associated target genes^30^. MED16, MED18 and CDK8 have all been shown to interact physically with MED25^86–88^. We found that stress-induced expression of a high proportion of JA-associated genes was dysregulated in *med9*, *med16* and *cdk8*. We hypothesize that JA-mediated stress signals in early phases of abiotic stress depend on interactions with several Mediator subunits, including MED9, MED16, MED25 and CDK8.

Evidence suggests that many stress-responsive genes exhibit diurnal or circadian oscillations^89^. Of 3,000 heat-responsive genes, ~70% showed time-of-day transcriptional response^90^. Conversely, mutations that affect chloroplast signals or diurnal rhythms lead to defects in abiotic stress responses^91^. Interestingly, we found that CDK8 is required for interaction with eight circadian clock TFs in early salt stress, which suggests that the CKM might function as a focal point linking the two processes.

MED18 interacts with several abiotic stress-response regulators. Our results corroborate the involvement of MED18 in the regulation of salt stress-responsive genes and suggest it may require a heterodimer of the C/S_1_ group bZIP TFs, bZIP11 and bZIP63. These TFs have been implicated as central regulators of the low-energy transcriptional response which may overlap with the abiotic stress response^92^, and the data presented here implicate MED18 as a co-regulator during salt stress. bZIP11 and MED18 have both been implicated in auxin-mediated transcription, suggesting a possible integration point for these signaling pathways^93,94^. Our results also identify a putative interaction between MED18 and light signaling pathways via the PIF1/PIF3 TFs.

The results of this analysis confirm previous reports implicating Mediator as a key regulator of abiotic stress responses in plants. We identify subunit-specific roles in different stresses and putative associations between individual subunits and TFs. Furthermore, our analyses suggest different modes of action for different Mediator subunits at the same loci. This provides information required for a deeper understanding of Mediator function in plants, as a prelude to biochemical characterization of the complex. We also provide a new perspective on the combinatorial nature of transcriptional regulation during stress, corroborating many prior results regarding the TF families involved in the wild-type abiotic stress response.

## Methods

### For full material and methods, please see supplemental information

#### Plant materials

All lines used were in the *Arabidopsis thaliana* Columbia (Col-0) background. Seeds of *med9* (SALK_029120), *med16* (alias *sfr6-2*; SALK_048091^24^) and *med18* mutants (SALK_027178^95^) were obtained from the Nottingham Arabidopsis Stock Centre (NASC; Nottingham, UK), and verified for T-DNA insertion and reduced transcript levels by RT-qPCR. The *cdk8* mutant (GABI_564F11) was described previously^39^.

#### Growth conditions and experimental treatments

Mature rosette plants (35 d) were grown in soil (heat and cold experiments) or in a hydroponic system adapted from^96^ (salt experiment) under short-day (8 h light: 16 h dark) conditions at 22 °C. Plants were sampled in control conditions (CON or CON_SS) prior to abiotic stress exposure. For heat stress, plants were incubated at 37 °C and sampled after 30 min (HS30) and 120 min (HS120). For cold stress, plants were incubated at 5 °C and sampled after 3 h (CS3) and 72 h (CS72). For the salt stress experiment, plants were sampled after 4 h (SS4) and 24 h (SS24) exposure to 200 mM NaCl. Four biological replicates were collected for each line and time-point and the expression of appropriate stress-specific marker genes was verified using RT-qPCR.

#### RNA isolation, RNA-seq library construction and sequencing

RNA was extracted from ~100 mg ground tissue using the E.Z.N.A Plant RNA kit (Omega Bio-tek, Norcross, USA) and genomic DNA removed using Turbo DNA*free* DNAse I (Ambion, Foster City, USA). RNA quantity and integrity was verified using an Agilent BioAnalyzer 2100 with RNA Nano 6000 kit (Agilent Technologies, Santa Clara, USA) prior to RNA-seq. Construction of cDNA libraries and RNA-seq was performed by the National Genomics Infrastructure (NGI; Uppsala, Sweden) after ribosomal RNA removal using Illumina Ribo-Zero rRNA removal kit (Plant Leaf). Single-end RNA-seq was performed on a HiSeq 2500 High Output V4 platform (Illumina, San Diego, USA), generating 13–32 million reads.

#### Pre-processing of RNA-seq data and identification of differentially expressed genes

Raw RNA-seq data was pre-processed by NGI Uppsala, according to best practice using TrimGalore and FastQC. Read counts were obtained using the kallisto R package (v0.43.0)^97^ and mapped to the Araport11 Arabidopsis Col-0 reference genome annotation^40^. Uniquely-mapping transcripts were counted and expressed as transcripts per million; overviews of our data are shown in Supplemental Table S9. A detected population of around 26,000 transcripts filtered for lowly-expressed transcripts generated 24,450, 24,194 and 25,914 genes for the cold, heat and salt stress experiments, respectively.

Transcriptomes were directly compared by PCA using the prcomp package, and a variance-stabilizing transformation (VST) was applied to the raw data using the Bioconductor DESeq2 package (v1.14.1,)^98^. Statistical analysis of gene and transcript differential expression (DE) between conditions was performed using DESeq2. Global gene expression was assumed to follow a negative-binomial distribution, and the thresholds for significant differential expression between genotypes or time-points were set at a Benjamini-Hochberg corrected *p*-value of 0.01 and an absolute log_2_ fold-change of 0.5^41^). GO and KEGG pathway enrichment analysis was analyzed using the GO function of Thalemine (v1.10.4; https://apps.araport.org/thalemine/bag.do), with the appropriate background of 24,454 genes and a Benjamini-Hochberg corrected *p-*value cut-off of 0.05 to account for multiple hypothesis testing.

### Gene co-expression network construction and analysis

A gene co-expression network was created using the program Seidr, which aggregates multiple networks generated using 11 different statistical inference methods, according to the toolkit documentation^99^. Briefly, all VST-normalized gene expression data sets were used as input for network construction. Included inference methods were ARACNE^100^, CLR^101^, Elastic Net and SVM ensemble^102^, Partial Correlation^103^, NARROMI^104^, GENIE3^105^, PLSNET^106^, Pearson, Spearman and TIGRESS^107^. The results were aggregated into a consensus network using the Top1 method and the network was filtered using a filter threshold of 0.999833. Infomap with a Markov-time set to 0.01 was used to detect modules of tightly connected (co-expressed) genes^108^. Data were visualized using the Infomap Navigator. A hypergeometric test was used to detect network modules enriched for gene-sets of interest (e.g. stress-responsive genes). We focused on the most significant stress modules using a *p*-value cut-off of 1E-5 and a size cut-off of at least 50 genes in the module.

### Transcription factor binding sites enrichment analysis

Enrichment of TFBS in the four stress regulons was analyzed using TF2Network^45^. A *p*-value of <0.01 was set as significance threshold. To correct for the smaller background population in our regulon analyses, a permutation test using 10,000 permutations and a significance level of *p* < 0.05 was used.

### Accession numbers

The sequencing data has been deposited at the European Nucleotide Archive (ENA, www.ebi.ac.uk/ena) under accession number PRJEB33339.

## Supplementary information

Supplementary Table S1.

Supplementary Table S2.

Supplementary Table S3.

Supplementary Table S4.

Supplementary Table S5.

Supplementary Table S6.

Supplementary Table S7.

Supplementary Table S8.

Supplementary Table S9.

Supplementary Table S10.

Supplementary Information.
