# Supplementary material for: Specific functions for Mediator complex subunits from different modules in the transcriptional response of Arabidopsis thaliana to abiotic stress
Source: Sci Rep. 2020 Mar 19;10:5073. doi: 10.1038/s41598-020-61758-w (PMC7081235; doi:10.1038/s41598-020-61758-w)
Supplement: Supplementary file 11 — Supplementary Information. [file 41598_2020_61758_MOESM11_ESM.pdf]

Supplemental Information

Specific functions for Mediator complex subunits from different modules in the transcriptional response of *Arabidopsis thaliana* to abiotic stress

Tim Crawford[a,c], Fazelaat Karamat[b1], Nóra Lehotai[a1], Matilda Rentoft[b1], Jeanette Blomberg[b], Åsa Strand[a], Stefan Björklund[b,2]

[a] Umeå Plant Science Centre, Department of Plant Physiology, Umeå University, Umeå 901 87, Sweden

[b] Department of Medical Biochemistry and Biophysics, Umeå University, Umeå 901 87, Sweden

[c] Present address: Institute for Biochemistry and Biology, University of Potsdam, Germany

[1]These authors contributed equally

[2]Corresponding author: stefan.bjorklund@umu.se

Contents

Supplemental Figures

Supplemental Figure S1……………………………………………… 3

Supplemental Figure S2……………………………………………… 4

Supplemental Figure S3……………………………………………… 5

Supplemental Figure S4……………………………………………… 6

Supplemental Figure S5……………………………………………… 7

Supplemental Figure S6……………………………………………… 8

Supplemental Figure S7……………………………………………… 9

Supplemental Figure S8……………………………………………… 10

Supplemental Figure Legends………………………………… 11-12

Supplemental Materials and Methods…………………………. 13-15

Supplemental References……………………………………… 16-17

Supplemental Table Legends…………………………………….. 18

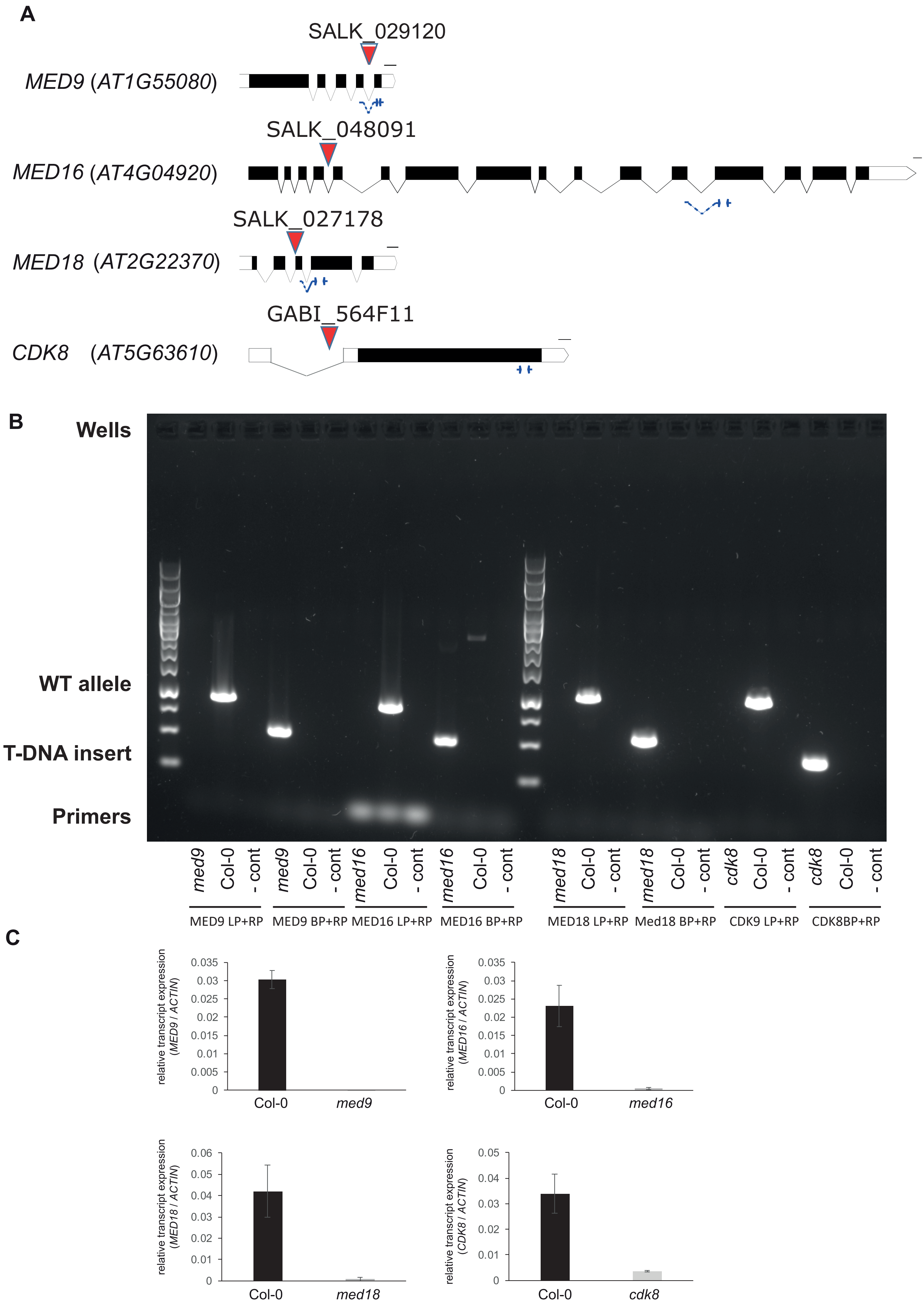

Supplemental Figure S1

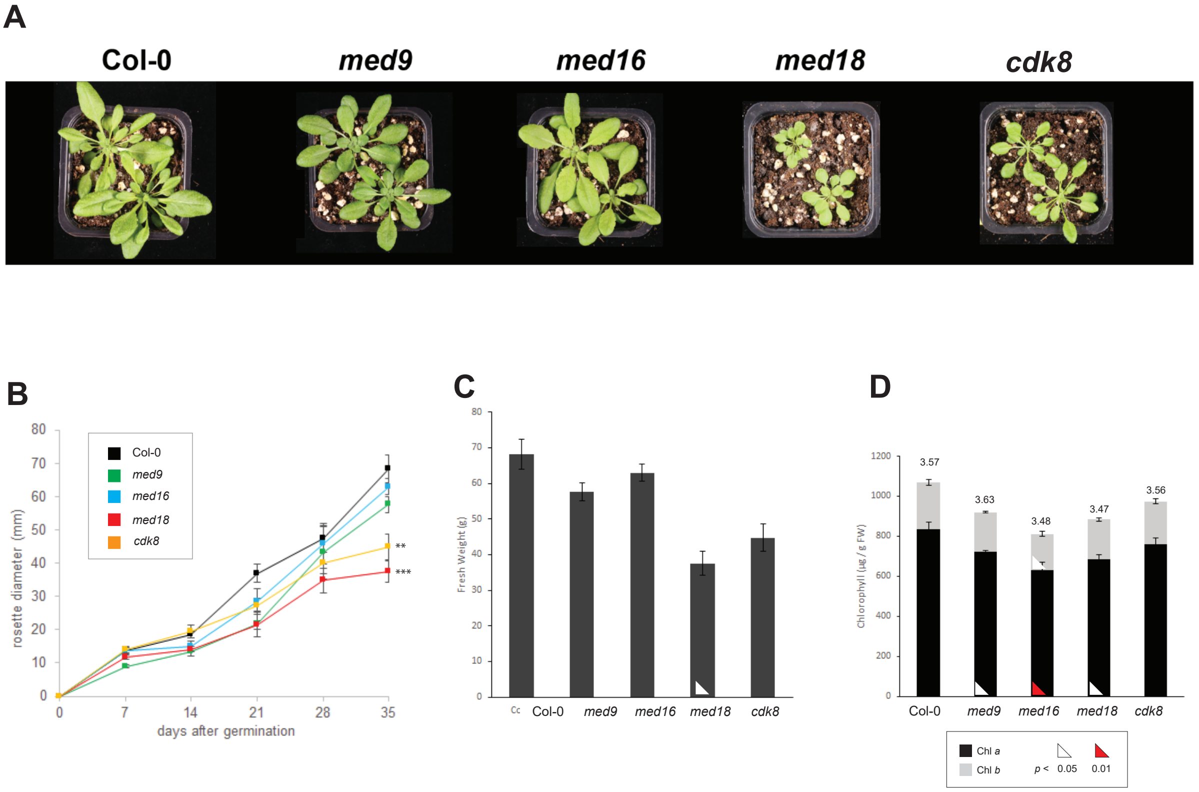

Supplemental Figure S2

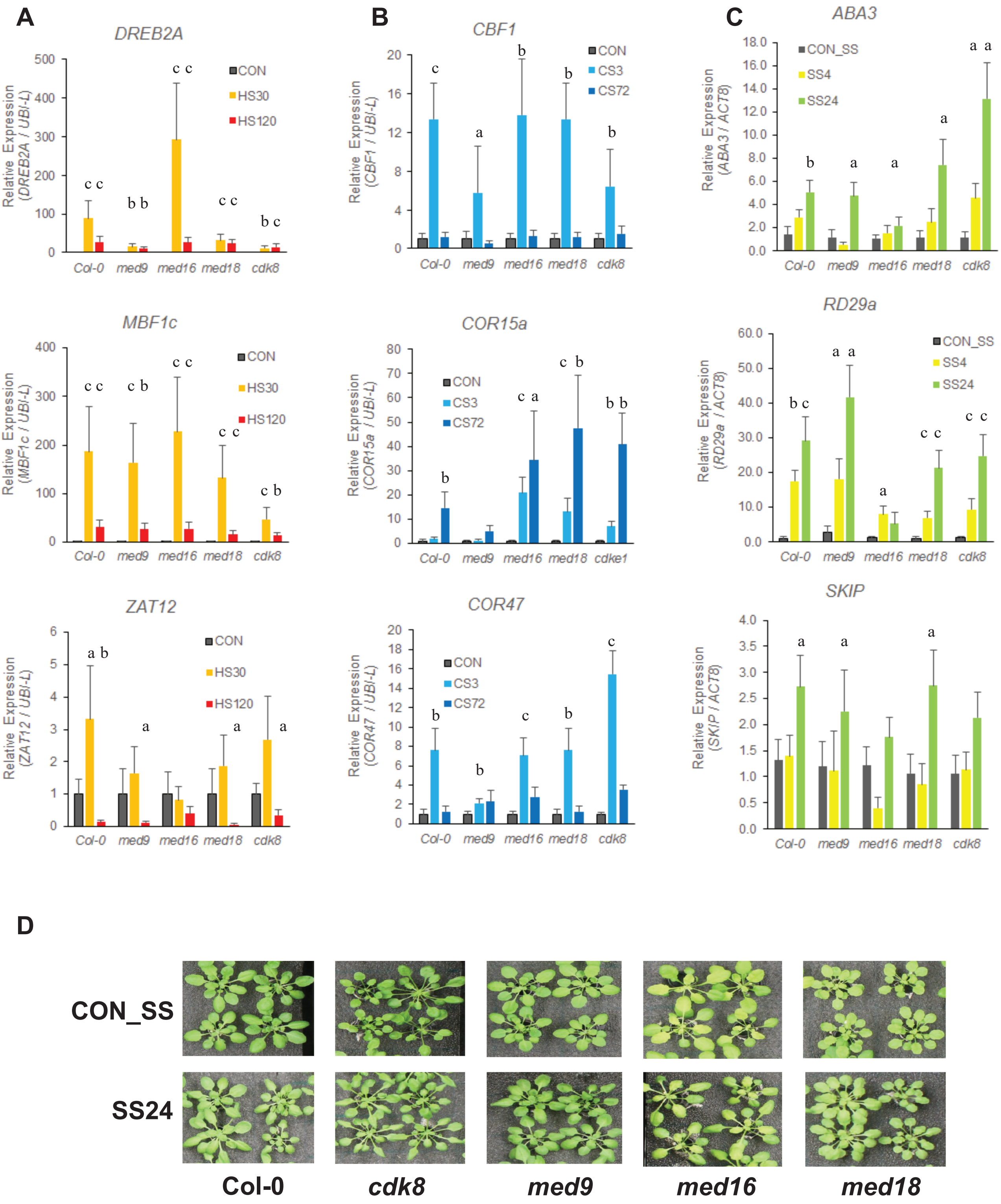
A
DREB2A
Relative Expression (DREB2A / UBI-L)
CON
HS30
HS120
Col-0 med9 med16 med18 cdk8
MBF1c
Relative Expression (MBF1c / UBI-L)
ZAT12
Relative Expression (ZAT12 / UBI-L)
B
CBF1
Relative Expression (CBF1 / UBI-L)
CON
CS3
CS72
COR15a
Relative Expression (COR15a / UBI-L)
Col-0 med9 med16 med18 cdke1
COR47
Relative Expression (COR47 / UBI-L)
C
ABA3
Relative Expression (ABA3 / ACT8)
CON_SS
SS4
SS24
RD29a
Relative Expression (RD29a / ACT8)
SKIP
Relative Expression (SKIP / ACT8)
D
CON_SS
SS24
Col-0
cdk8
med9
med16
med18

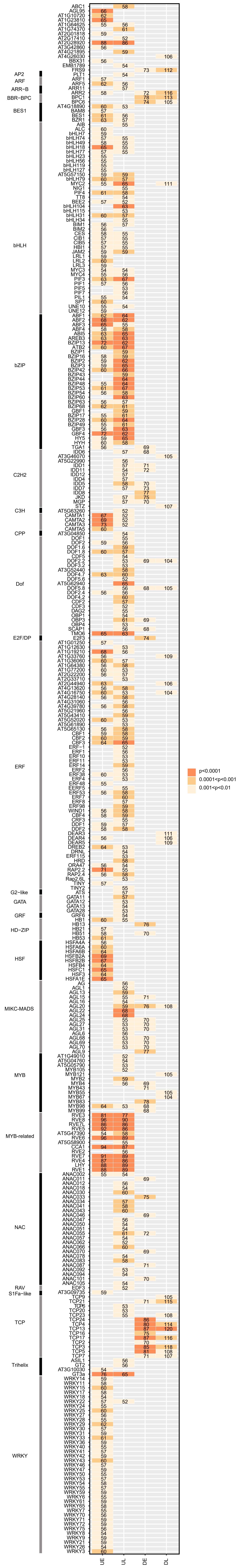

Supplemental Figure S4

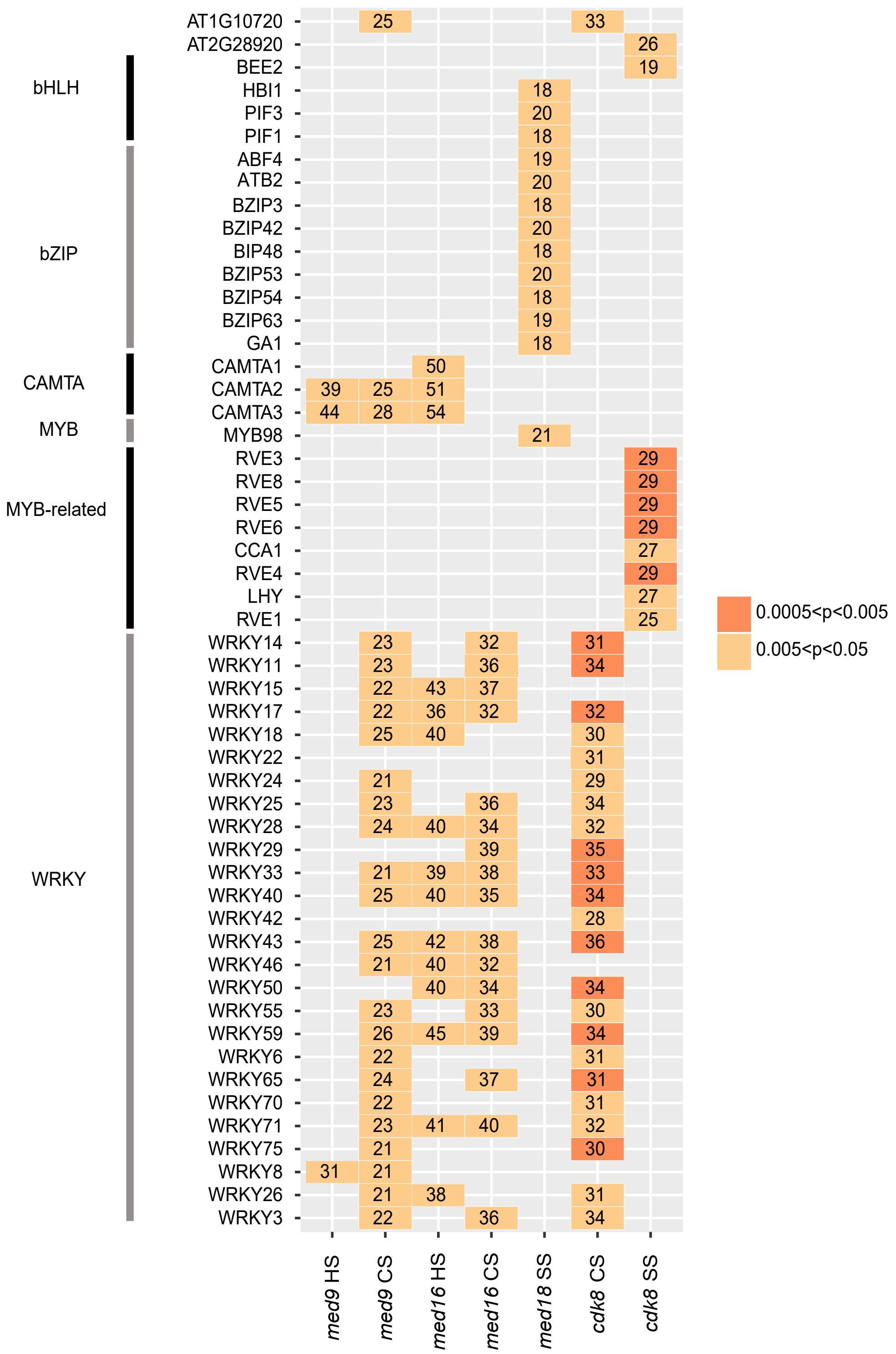

Supplemental Figure S5

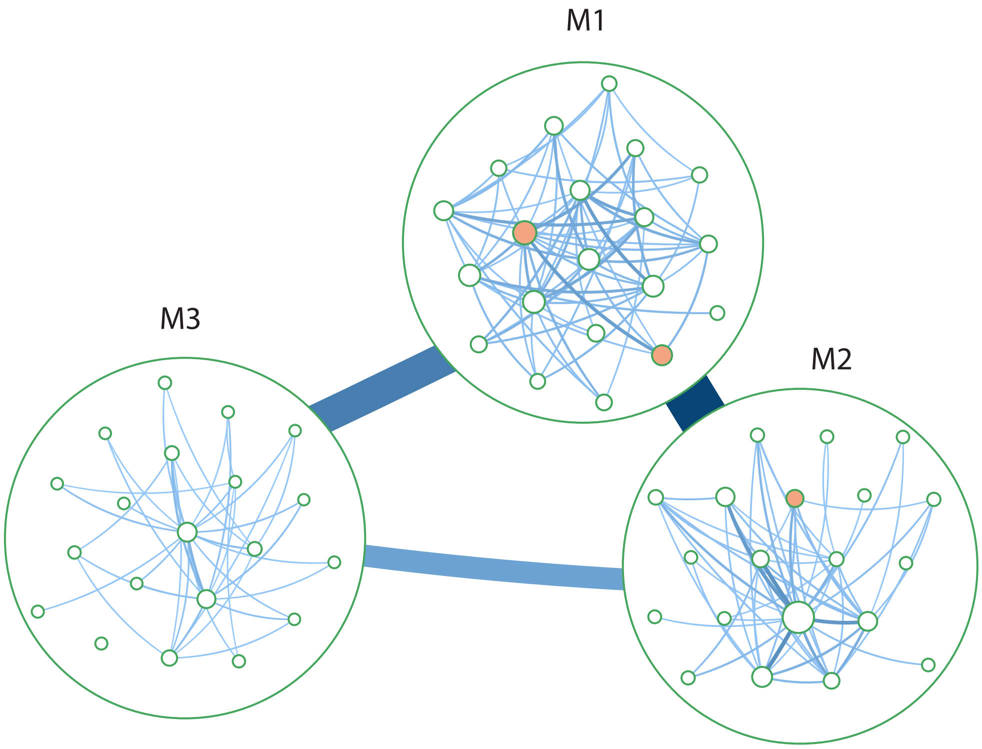

Supplemental Figure S6

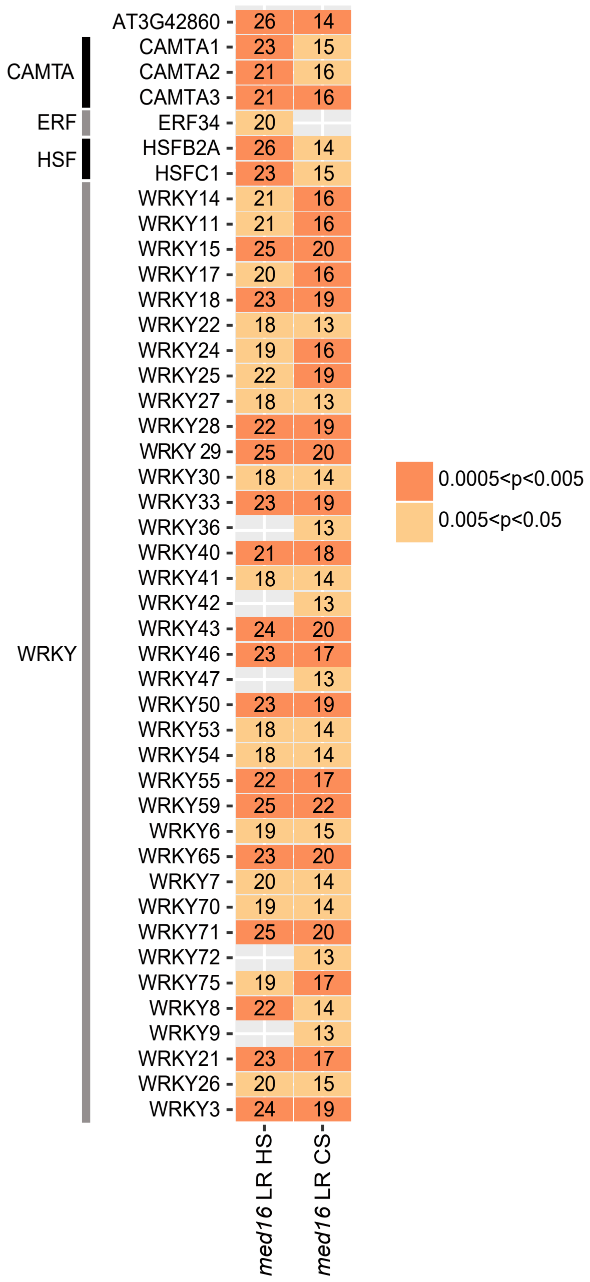

Supplemental Figure S7

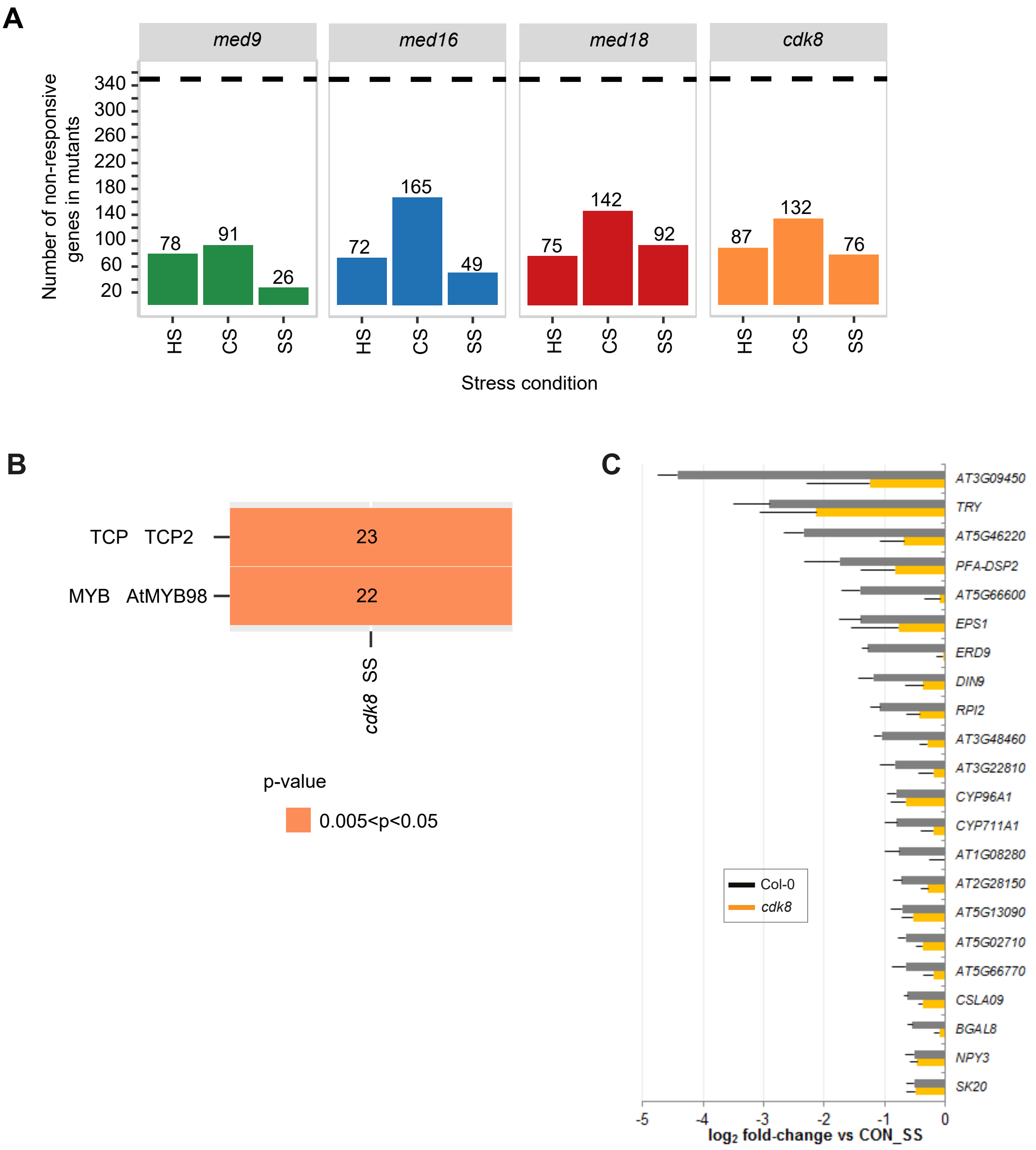

Supplemental Figure S8

Supplemental Figure S1. Genotyping and verification of Mediator mutant lines used in this study. (A) Genomic diagrams illustrating the structure of the *MED9*, *MED16, MED18* and *CDK8* genes and position of the T-DNA insert in the indicated SALK or GABI mutant lines. Protein-coding regions are indicated in black and untranslated regions in white, and the small black bars indicate 100 nt. Blue lines and arrowheads indicate locations for primers used in RT-qPCR. Dashed blue lines indicate nucleotides that are not included in the primer when it includes parts of two exons. (B) Verification of mutant genotypes and homozygosity. Genomic DNA was isolated from the wild type and mutant lines, and PCR was performed using gene specific Left boarder (LP) and Right boarder (RP) primers for r the wild-type allele (~1000 nt) or gene specific Right boarder (RP) together with the LBb3.1 (BP) primer specific for the T-DNA insert (~500 nt). "- cont" represents the negative control with water. The LP, RP and Lb3.1 are all standard primers from the Salk Institute found at signal.salk.edu/tdnaprimers.2.html (C) RT-qPCR verification of transcript depletion in mutant lines. RNA was isolated from Col-0 and plants from each mutant line, and RT-qPCR performed using the gene-specific primers illustrated in blue. Data shown are the expression levels of each transcript relative to the reference gene, actin (*AT2G37620*), n=3 biological replicates. Sequences of the primers used for these analyses are listed in Supplemental Table S10.

Supplemental Figure S2. Rosette phenotype of *Arabidopsis* Col-0 and Mediator mutant lines used in this study. (A) Photographs of Col-0 and Mediator mutant plants, grown on soil for 35d under (short-day) greenhouse control conditions. (B) Rosette diameter of Col-0 and Mediator mutant plants during growth in short-day conditions. Rosettes were measured at noon, across the widest point. Col-0 wild type: black; *med9*: green; *med16*: cyan; *med18*: red; *cdk8*: orange. (C) Biomass (fresh weight) and (D) chlorophyll content of mature rosette plants at 35d. Data shown are the mean ± the standard error from at least 20 individual plants. Two or three asterisks, or white or red triangles, indicate a *p*-value of less than 0.05 or 0.01, respectively (calculated in comparison with Col-0). Numbers above bars in (D) indicate the chlorophyll *a*:*b* ratio.

Supplemental Figure S3. Verification of abiotic stress conditions and time-point selection by RT-qPCR. Selected genes were used as markers for the transcriptional response to (A) heat stress, (B) cold stress or (C) salt stress. Data shown are the mean expression relative to the expression of a reference gene (*UBI-L* for heat and cold or *ACTIN8* for salt stress) and to the expression level in CON or CON_SS for each genotype. Error bars indicate the standard error (n = 4). P values are calculated based on the ΔC_t values a, P ≤ 0.05; b, P ≤ 0.01; c, P ≤ 0.001 (D) Photographs of hydroponic-grown wild type and mutant lines before (Control) and at the late (SS24) stage of salt stress treatment.

Supplemental Figure S4. Enrichment of regulatory *cis*-elements in the promoters of genes in the four common abiotic stress regulons. Enrichment analysis of transcription factor (TF) binding sites using TF2Network in EARLY UP (EU), LATE UP (LU), EARLY DOWN (ED) and LATE DOWN (LD) regulons. Numbers within rectangles indicate the number of genes containing a binding site for the corresponding TF. Darker shades of orange indicate increasing enrichment of TF binding sites.

Supplemental Figure S5. Enrichment of regulatory *cis*-elements in the promoters of non-responsive genes within the EARLY UP common abiotic stress regulon in Mediator mutants. Enrichment analysis of transcription factor (TF) binding sites in non-responsive genes in the Mediator mutants using TF2Network, as compared to the full set of 281 transcripts in the EARLY UP regulon. Numbers within rectangles indicate the number of genes containing a binding site for the corresponding TF. Darker shades of orange indicate increasing enrichment of TF binding sites.

Supplemental Figure S6. Co-expression network visualizing the 20 largest modules at the first and second level. Modules significantly enriched for EARLY UP gens are colored beige.

Supplemental Figure S7. Enrichment of regulatory *cis*-elements in the promoters of non-responsive genes of the EARLY UP common abiotic stress regulon which displayed loss of

repression in *med16*. Enrichment analysis of transcription factor (TF) binding sites in non-responsive genes displaying loss of repression (LR) prior to stress in the *med16* mutant using TF2Network, as compared to the full set of 281 transcripts in the EARLY UP regulon. Numbers within rectangles indicate the number of genes containing a binding site for the corresponding TF. Darker shades of orange indicate increasing enrichment of TF binding sites.

Supplemental Figure S8. Non-responsive genes of the EARLY DOWN common abiotic stress regulon in Mediator mutants. (A) Bar graph of the numbers of genes of the EARLY DOWN regulon which were non-responsive (not significantly down-regulated) in the Mediator mutants. The dotted line indicates the total number of genes in the regulon (349). (B) Enrichment analysis of transcription factor (TF) binding sites in non-responsive genes in the Mediator mutants using TF2Network, as compared to the full set of 349 transcripts in the EARLY DOWN regulon, summarised to the level of TF-families. Numbers within rectangles indicate the number of genes containing a binding site for the corresponding TF. (C) Bar plot of salt stress-induced expression changes in selected genes containing TF binding sites for TCP2 and MYB98 in Col-0 wild type (black) and the *cdk8* mutant (orange). Data shown are the mean $\log_2$ fold-change in expression for each transcript at SS4 relative to the level at CON_SS ± SD (*n*=4).

SUPPLEMENTAL EXPERIMENTAL PRODECURES

Plant Materials

All plant lines used in the study were in the *Arabidopsis thaliana* Columbia (Col-0) background. Seeds of the *med9* (SALK_029120), *med16* (alias *sfr6-2*; SALK_048091;[1] and *med18* mutants (SALK_027178;[2] were obtained from the Nottingham Arabidopsis Stock Centre (NASC; Nottingham, UK). The *cdk8* mutant (GABI_564F11) has been described previously [3]. All Mediator knock-out lines were verified as homozygous for the appropriate T-DNA insertion and transcript levels were quantified by RT-qPCR using Roche 454 (Roche, Clifton NJ, USA). The primers used for genotyping and RT-qPCR checks are listed in Supplemental Table S10. Biomass was calculated as the fresh weight of aerial parts of at least 20 individual plants, and chlorophyll content in acetone extracts from leaves of the same plants was determined according to [4] and normalized to the fresh weight.

Growth Conditions and Experimental Treatments

Seeds of the different *Arabidopsis* plant lines were surface sterilized and plated on 1×MS medium supplemented with 1% (w/v) sucrose and kept for 3 d at 4°C in darkness for stratification of the seeds. After 7 d growth on plates under long-day greenhouse conditions (16 h light / 8 h dark photoperiod, 22°C/18°C day/night temperature and 65% humidity), seedlings were transferred to soil-filled pots and grown under short-day greenhouse (control) conditions (22°C/18°C day/night temperature and 65% humidity) under standard illumination (cool white light provided by fluorescent tubes (Osram Lumilux L18W 840 (Germany); peak wavelength 840 nm) at a photon flux density of 120 $\mu E.m^{-2}.s^{-1}$ in an 8 h light / 16 h dark photoperiod) for a further 28 d.

After totally 35 d growth, rosette plants were exposed to abiotic stress conditions, beginning 2 h into the light period. Plants were first photographed and sampled in control conditions (CON). For heat stress (HS), plants were transferred to a heat chamber (Stuart Incubator SI60D, Staffordshire, UK) maintained at 37°C with standard illumination and 65% humidity and sampled after 30 min (HS30) and 120 min (HS120). For cold stress (CS), plants were transferred to a cold room maintained at 5°C, with standard illumination and 67% humidity, and sampled after 3 h (CS3) and 72 h (CS72). In both cases, temperatures were verified and monitored using one internal (system) and one external thermometer. These temperatures were chosen as they are known to be non-lethal for *Arabidopsis,* but enough to induce a transcriptional response; the time-points for sampling were empirically determined during protocol optimization (with guidance from the literature) to encompass peaks in transcriptional activity (see Fig S3).

For the salt stress (SS) experiment, a second population of Col-0 (wild-type) and mutant plants were grown in hydroponic system adapted from [5], and liquid and solid media used in the system were prepared as described therein. Seeds were surface sterilized, and 2-3 seeds placed in solid germination media in a black Eppendorf tube. These tubes were placed in liquid germination media in a 24-well floating microtube rack with hinged lid under standard illumination and short-day greenhouse control conditions. After 7 d, seedlings were thinned to one per Eppendorf tube, liquid germination media was changed to liquid basal nutrient solution, and lid was replaced with cling-film. After 21 d, plants were transferred from boxes to aerated tanks supplied with basal nutrient solution. After 35 d, plants were exposed to salt stress by replacing the media with freshly prepared basal nutrient solution supplemented with 200 mM of NaCl; for control samples, media was replaced with fresh un-supplemented basal nutrient solution. Samples for RNA-seq were collected before (CON_SS) and after 4 h (SS4) and 24 h (SS24) exposure to salt, which began 2 h into the light period.

Four biological replicates were collected for each line and time-point (where each biological replicate consisted of one rosette leaf each from six individual plants), flash-frozen in liquid N_2 and stored at -80°C. Collection of samples was completed within 5 minutes. In order to verify that our experimental

conditions resulted in induction of an appropriate biological stress response, and that we were sampling at time-points that would encompass peaks in transcriptional activity, stress-specific marker genes were selected for RT-qPCR measurement as described [6] using a BioRad CFX 96 (Hercules, USA) and/or Roche 454 (Clifton NJ, USA). For heat stress, these marker genes included *DREB2a, MBF1c and ZAT12* [7]*;* for the cold stress, *CBF1, COR15a* and *COR47* [6]; and for salt stress, *ABA3, RD29A* and *SKIP* [8]. For the heat and cold stress experiments, *ubiquitin-like* (*AT4G36800*) was used as the reference gene, while for the salt stress we used *actin 8* (*AT1G49240*). The oligonucleotide primer sequences used for these RT-qPCR analyses are listed in Supplemental Table S10.

RNA Isolation, RNA-Seq Library Construction and Sequencing

For RNA isolation, tissue was ground to fine powder using a mortar and pestle cooled in liquid N_2 and RNA was extracted from ~100 mg tissue using the E.Z.N.A Plant RNA kit (Omega Bio-tek, Norcross, USA). To remove DNA contamination, samples were treated with Turbo DNA*free* DNAse I (Ambion, Foster City, USA) at 37°C for 45 minutes, followed by inactivation as per the manufacturer's instructions. The quantity and integrity of RNA samples were verified using an Agilent BioAnalyzer 2100 with RNA Nano 6000 kit (Agilent Technologies, Santa Clara, USA), and only samples with an RNA integrity number ≥8 were sent for RNA-seq.

Verified RNA samples were submitted to the National Genomics Infrastructure (NGI; Uppsala, Sweden) for cDNA library construction and sequencing. The Illumina Ribo-Zero rRNA removal kit (Plant Leaf) was used to remove rRNA contamination, and cDNA library construction was performed. All samples were dual barcoded, multiplexed and loaded onto ten sequencing lanes. Single-ended 50 bp sequencing was performed on a HiSeq 2500 High Output V4 platform (Illumina, San Diego, USA), generating between 13 and 32 million reads.

Pre-processing of RNA-Seq data and Identification of Differentially Expressed Genes

Raw RNA-seq data was pre-processed by NGI Uppsala, according to best practice described in https://ngisweden.scilifelab.se/file/New%20RNA-seq%20Bioinformatics%20Best%20Practice. Briefly, FastQC (v0.11.4; http://www.bioinformatics.babraham.ac.uk/projects/fastqc/) was used for quality control of the raw data. The data were further filtered to remove adapters and trimmed for quality using Trim Galore (https://www.bioinformatics.babraham.ac.uk/projects/trim_galore/). Filtered reads went through FastQC check again to guarantee that there were no technical artefacts generated. The raw data was double-checked by the authors, using a another, previously developed pipeline for quality control, read mapping and expression quantification [9,10], following the guidelines described in http://www.epigenesys.eu/en/protocols/bio-informatics/1283-guidelines-for-rna-seq-data-analysis. The data generated by the two pipelines were in agreement, so we proceeded using the original NGI-generated raw data.

Read counts were obtained using the kallisto R package (v0.43.0, [11]) with the parameters quant -b 100 -t --rf-stranded and mapped to a custom-made GFF3 file comprising both the coding and non-coding RNA (ncRNA) features of the Araport11 Arabidopsis Col-0 reference genome annotation [12]. The code was executed as follows: kallisto quant -i kallisto/Araport11_all.201606.cdna.inx -b 100 -o kallisto/P6960_144_R1_trimmed --rf-stranded -t 8 --rf-stranded trim_galore/P6960_144_R1_trimmed.fq.gz -l 250 -s 50. Uniquely-mapping transcripts were counted and expressed as transcripts per million. Overviews of our data, including raw read counts, are shown in Supplemental Table S9. The kallisto abundance values were imported into R (v3.3.2; R Core Team 2015) with the help of the Bioconductor (v3.3; [13]) tximport package (v.1.2.0; [14]). Beginning with a detected population of around 26,000 out of 32,834 annotated features such as genes and ncRNAs, lowly-expressed transcripts (less than one transcript, in no more than 2 of 4 replicates in any condition) were filtered out using a custom feature-select script to produce 24,450, 24,194 and 25,914 genes for the cold, heat and salt stress experiments, respectively. Direct comparison of global transcriptomes was achieved

with Principal Component Analysis (PCA), performed using the R core package prcomp, or by creating heatmaps using custom R scripts. To normalize the data and enable better comparison between highly- and lowly-expressed genes, a variance-stabilizing transformation was applied on the raw data using the Bioconductor DESeq2 package (v1.14.1; [15]). Clustering and scaling of variance-stabilized data was performed using Ward's method of Pearson correlation. Heatmaps, PCA figures, bubble plots and box-and-whisker plots were generated in R using the ggplot2 package (v2.3.0).

Statistical analysis of gene and transcript differential expression (DE) between conditions was performed in R using DESeq2 with the following model: ~genotype*time-point, to account for all the genotypes (Col-0 wild type, *med9*, *med16*, *med18* and *cdk8*) and all time-points in each stress conditions (CON, EARLY and LATE). Global gene expression was assumed to follow a negative-binomial distribution, and the thresholds for significant differential expression between genotypes or time-points were set at a Benjamini-Hochberg corrected *p*-value of 0.01 and an absolute $\log_2$ fold-change of 0.5 (following the recommendations of [16] for DESeq2 with four biological replicates). All the expression results were generated in R, using custom scripts.

Gene Ontology Functional Enrichment Analyses

Gene ontology (GO) and Kyoto Encylopedia of Genes and Genomes (KEGG) pathway enrichment analysis was analyzed using either: the GO function of Thalemine (v1.10.4; https://apps.araport.org/thalemine/bag.do), with the appropriate background of 24,454 genes and a Benjamini-Hochberg corrected *p*-value cut-off of 0.05 to account for multiple hypothesis testing; or the integrated Enrichment function of AtGenie (www.atgenie.org) [10]. Lists of enriched GO functional categories were reduced and redundant categories removed using REVIGO (http://revigo.irb.hr/; [17]) with default settings but for the database that was set to "Arabidopsis thaliana". Complete sets of associated Gene IDs for each GO functional category were obtained from AmiGO (http://amigo.geneontology.org/amigo/) and manually curated. Overlaps between gene sets were determined using Venny (v2.1; [18].

Transcription Factor Binding Site Enrichment Analysis

Enrichment of transcription factor (TF) binding sites in the four stress regulons was analyzed using TF2Network [19]. A *p*-value of < 0.01 was set as the threshold for significance. The output file from TF2Network generally contained multiple binding sites for the same TF. To increase interpretability in figures, data were collapsed to the level of TFs or TF-families, which we defined according to the Arabidopsis specific file from PlantTFDB [20]. To test for enrichment of any of TF binding sites in the subgroup of the stress regulon genes which did not respond to stress in the mutants ('non-responsive'), the number of targets (genes with TF binding site) in the subgroup 'non-responsive' was compared to the total number of targets in the respective stress regulon. For this, a permutation test using 10,000 permutations and a significance level of $p < 0.05$ was used.

Accession Numbers and Data Access

The sequencing data has been deposited at the European Nucleotide Archive (ENA, www.ebi.ac.uk/ena) under accession number PRJEB33339.

REFERENCES FOR SUPPLEMENTAL MATERIALS AND METHODS

1. Knight, H. *et al.* Identification of SFR6, a key component in cold acclimation acting post-translationally on CBF function. *Plant J.* **58**, 97–108 (2009).

2. Kim, Y. J. *et al.* The role of Mediator in small and long noncoding RNA production in *Arabidopsis thaliana*. *EMBO J.* **30**, 814–822 (2011).

3. Ng, S. *et al.* Cyclin-dependent kinase E1 (CDKE1) provides a cellular switch in plants between growth and stress responses. *J. Biol. Chem.* **288**, 3449–3459 (2013).

4. Porra, R. J., Thompson, W. A. & Kriedemann, P. E. Determination of accurate extinction coefficients and simultaneous equations for assaying chlorophylls a and b with four different solvents: verification of the concentration of chlorophyll standards by atomic absorption spectroscopy. *Biochim. Biophys. Acta* **975**, 384–394 (1989).

5. Conn, S. J. *et al.* Protocol: optimising hydroponic growth systems for nutritional and physiological analysis of Arabidopsis thaliana and other plants. *Plant Methods* **9**, 1–11 (2013).

6. Noren, L. *et al.* Circadian and plastid signaling pathways are integrated to ensure correct expression of the CBF and COR genes during photoperiodic growth. *Plant Physiol.* **171**, pp.00374.2016 (2016).

7. Ohama, N., Sato, H., Shinozaki, K. & Yamaguchi-Shinozaki, K. Transcriptional Regulatory Network of Plant Heat Stress Response. *Trends Plant Sci.* **22**, 53–65 (2017).

8. Lim, G. H. *et al.* A putative novel transcription factor, AtSKIP, is involved in abscisic acid signalling and confers salt and osmotic tolerance in Arabidopsis. *New Phytol.* **185**, 103–113 (2010).

9. Delhomme, N. *et al.* Guidelines for RNA-Seq data analysis. *Epigenesys* 1–24 (2014).

10. Sundell, D. *et al.* AspWood: High-Spatial-Resolution Transcriptome Profiles Reveal Uncharacterized Modularity of Wood Formation in *Populus tremula*. *Plant Cell* **29**, 1585–1604 (2017).

11. Bray, N. L., Pimentel, H., Melsted, P. & Pachter, L. Near-optimal probabilistic RNA-seq quantification. *Nat. Biotechnol.* **34**, 525–527 (2016).

12. Cheng, C. Y. *et al.* Araport11: a complete reannotation of the Arabidopsis thaliana reference genome. *Plant J.* **89**, 789–804 (2017).

13. Gentleman, R. C. *et al.* Bioconductor: open software development for computational biology and bioinformatics. *Genome Biol.* **5**, R80 (2004).

14. Soneson, C., Love, M. I. & Robinson, M. D. Differential analyses for RNA-seq: transcript-level estimates improve gene-level inferences. *F1000 Res.* **4**, 1–18 (2018).

15. Love, M. I., Huber, W. & Anders, S. Moderated estimation of fold change and dispersion for RNA-seq data with DESeq2. *Genome Biol.* **15**, 1–21 (2014).

16. Schurch, N. J. *et al.* How many biological replicates are needed in an RNA-seq experiment and which differential expression tool should you use? *RNA* **22**, 1641–1641 (2016).

17. Supek, F., Bošnjak, M., Škunca, N. & Šmuc, T. Revigo summarizes and visualizes long lists of

gene ontology terms. *PLoS One* **6**, 62–65 (2011).

18. Oliveros, J. C. Venny: An interactive tool for comparing lists with Venn's diagrams. (2007). Available at: http://bioinfogp.cnb.csic.es/tools/venny/index.html.

19. Kulkarni, S. R., Vaneechoutte, D., Van de Velde, J. & Vandepoele, K. TF2Network: predicting transcription factor regulators and gene regulatory networks in Arabidopsis using publicly available binding site information. *Nucleic Acids Res.* **46**, e31–e31 (2017).

20. Jin, J. *et al.* PlantTFDB 4.0 : toward a central hub for transcription factors and regulatory interactions in plants. *Nucleic Acids Res.* **45**, 1040–1045 (2017).

SUPPLEMENTAL TABLE LEGENDS

Supplemental Table S1. Differentially expressed genes (DEGs) in Mediator mutants compared to Col-0 wild type.
Supplemental Table S2. Clusters of co-expressed transcripts in the response of Col-0 wild type to abiotic stress.
Supplemental Table S3. DEGs in the response of Col-0 wild type to abiotic stress.
Supplemental Table S4. Partitioned gene co-expression network file in .ftree format, and a key to translate Araport GeneIDs into network nodes (for more information see https://www.mapequation.org/).
Supplemental Table S5. Modules in the gene co-expression network that are significantly enriched for a gene-set of interest. A corrected *p*-value of 1E-05 and a module size of above 50 were used as cut-offs.
Supplemental Table S6. DEGs in the common abiotic stress regulons and their expression in Col-0 wild type during abiotic stress.
Supplemental Table S7. Responses in Mediator mutants for each of the DEGs in the four common Col-0 stress regulons.
Supplemental Table S8. Output from TF2network analysis of all 390 genes in co-expression network module M1:7.
Supplemental Table S9. Raw read counts from RNA-sequencing for the Col-0 wild type and Mediator mutants during abiotic stress.
Supplemental Table S10. Oligonucleotide primers used in this study.
